# Training Children to Perceive Non-native Lexical Tones: Tone Language Background, Bilingualism, and Auditory-Visual Information

**DOI:** 10.3389/fpsyg.2018.01508

**Published:** 2018-09-04

**Authors:** Benjawan Kasisopa, Lamya El-Khoury Antonios, Allard Jongman, Joan A. Sereno, Denis Burnham

**Affiliations:** ^1^MARCS Institute for Brain, Behaviour and Development, Western Sydney University, Penrith, NSW, Australia; ^2^Department of Linguistics, College of Liberal Arts & Sciences, University of Kansas, Lawrence, KS, United States; ^3^Phonetics & Psycholinguistics Laboratory, Department of Linguistics, College of Liberal Arts & Sciences, University of Kansas, Lawrence, KS, United States

**Keywords:** lexical tone, auditory-visual, speech perception, bilingualism, perceptual attunement

## Abstract

This study investigates the role of language background and bilingual status in the perception of foreign lexical tones. Eight groups of participants, consisting of children of 6 and 8 years from one of four language background (tone or non-tone) × bilingual status (monolingual or bilingual)—Thai monolingual, English monolingual, English-Thai bilingual, and English-Arabic bilingual were trained to perceive the four Mandarin lexical tones. Half the children in each of these eight groups were given auditory-only (AO) training and half auditory-visual (AV) training. In each group Mandarin tone identification was tested before and after (pre- and post-) training with both auditory-only test (ao-test) and auditory-visual test (av test). The effect of training on Mandarin tone identification was minimal for 6-year-olds. On the other hand, 8-year-olds, particularly those with tone language experience showed greater pre- to post-training improvement, and this was best indexed by ao-test trials. Bilingual vs. monolingual background did not facilitate overall improvement due to training, but it did modulate the efficacy of the Training mode: for bilinguals both AO and AV training, and especially AO, resulted in performance gain; but for monolinguals training was most effective with AV stimuli. Again this effect was best indexed by ao-test trials. These results suggest that tone language experience, be it monolingual or bilingual, is a strong predictor of learning unfamiliar tones; that monolinguals learn best from AV training trials and bilinguals from AO training trials; and that there is no metalinguistic advantage due to bilingualism in learning to perceive lexical tones.

## Introduction

Like consonants and vowels, lexical tone is subject to perceptual attunement as a product of specific language experience. However, unlike consonants and vowels, lexical tone is not used to distinguish meaning in all the languages of the world. While tone languages comprise 70% of the world's languages (Yip, 2002) and more than 50% of the world's population speak a tone language (Fromkin, 1978), one of the most prevalent world languages, English, and one that has hosted the vast majority of language development studies, is not a tone language. On the other hand another of the most prevalent languages, Mandarin, is a tone language. This paper concerns training 6- and 8-year-old children to perceive novel lexical tones and whether such training is assisted by visual information for tone, previous tone language experience, and bilingual vs. monolingual experience. The experimental study is prefaced by exposition of the nature of lexical tone, attunement to lexical tone in infancy and in school-aged children, tone perception in monolingual and bilingual populations, and perceptual training methods for children.

### Lexical tone

**Figure 1:**
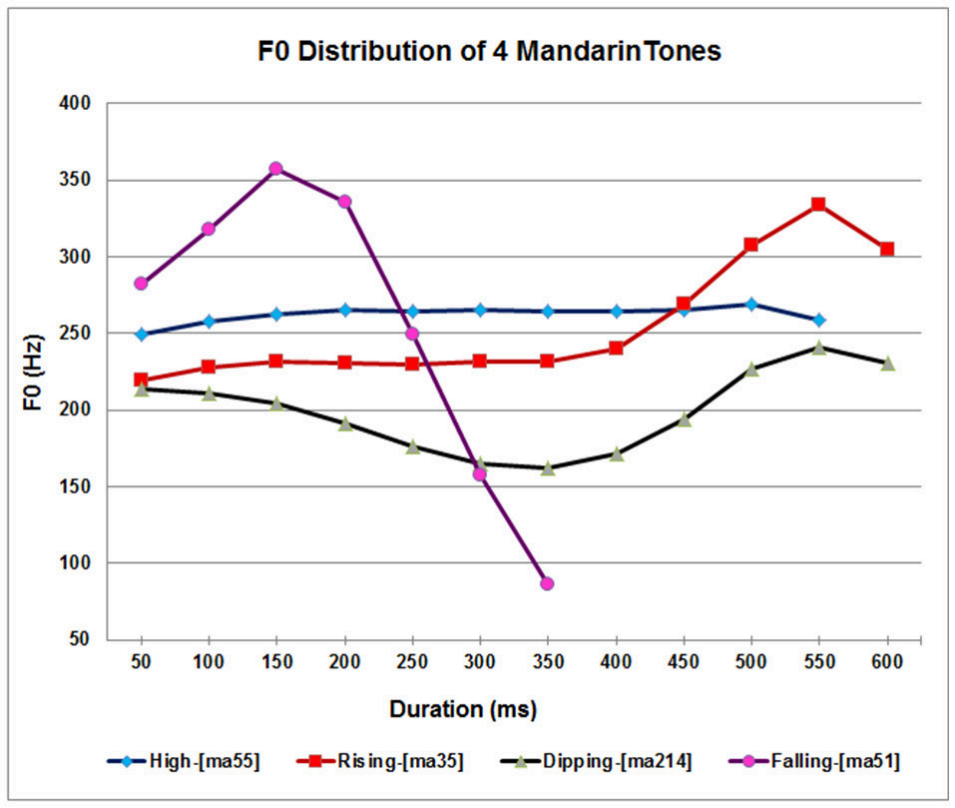
Fundamental frequency (F0) plots over time for the four Mandarin tones on the syllable /ma/ meaning “mother” [ma55]; “hemp”[ma35]; “horse”[ma214]; and “scold”[ma51] produced by four female native speakers of Beijing Mandarin dialect.

Lexical tone is a linguistic device contributing to the semantic realization of words. The main cue for lexical tone is fundamental frequency, perceived as pitch, but lexical tone is also characterized by variations in amplitude, duration and voice quality (Yip, 2002). Tones vary in type, level/static or contour/dynamic, with small or rapid pitch variation over time, respectively (Abramson, 1978). Tone languages also vary in the number of tones: for example, Thai has five tones, three level and two contour, Cantonese has three level and three contour tones, and Mandarin, the tone language to be investigated here, has one level and three contour tones (Yip, 2002). Figure 1 shows the pitch patterns over time of the four Mandarin tones and the corresponding meanings when spoken on the syllable /ma/. Tone 1 is a High-Level [T55^1^] tone; and tones 2, 3 and 4 are contour tones identified as a Mid-Rising [T35], Low-Falling-Rising [T214], and High-Falling [T51] (Hallé et al., 2004).

Tones 2 and 3 are the most difficult to perceive for first language (L1) children and second language (L2) adults. For example, Wong et al. (2005) found that 3-year-old Mandarin speaking children can accurately identify Tones 1, 2, and 4, but often confuse Tone 2 with Tone 3, and Li and Thompson (1977) showed that Mandarin-speaking children acquire Tones 2 and 3 later than Tones 1 and 4. Nevertheless, discrimination of the difficult Tones 2 and 3 improves dramatically after training, whereas the more easily discriminated Tones 1 and 4 are relatively resistant to improvement (Wang et al., 1999; Smith and Burnham, 2012).

### Perceptual attunement in infancy

Difficulties in discrimination of non-native language sounds by L2 tone language learners would appear to be related to perceptual attunement in infancy. Newborn infants perceive both native and non-native speech contrasts in a similar manner, but this language-general speech perception becomes more language-specific over infants' first year—there is a decline in discrimination performance for non-native speech contrasts while that for native speech contrasts is maintained or improves (Burnham and Mattock, 2010). Such perceptual attunement is evident between 7 and 11 months for consonants (Werker and Tees, 1984), between 4 and 6 months for vowels (Polka and Werker, 1994), and around the same age for lexical tones. Mattock and Burnham (2006), testing tone-language and non-tone-language-environment infants found that between 6 and 9-month-old English language infants' discrimination performance declined for Thai tones, but not for violin sounds created to have the same F0 contours as the tones, whereas Chinese infants showed no decline for either. Together, with similar studies with English and French infants (Mattock et al., 2008), these results show perceptual attunement for lexical tones that is specific to speech. More recently, Yeung et al. (2013) found distinctly different patterns of Cantonese tone perception at 4 months between Cantonese infants (for whom the tones were native), Mandarin infants (tones non-native), and English non-tone language infants. These results suggest that there is perceptual attunement for lexical tones by at least 4 months of age even before that for consonants and vowels (but see also Choi et al., 2017).

### Perceptual attunement in childhood

While there is perceptual attunement in infancy toward native and away from non-native sounds, non-native sounds are still perceivable and especially so under certain conditions (e.g., Werker and Logan, 1985), otherwise L2 learning would not be possible. Over and above this residual ability to perceive non-native sounds, there is now known to be a second period of perceptual attunement at the onset of reading for both consonants (Burnham et al., 1991; Burnham, 2003; Horlyck et al., 2012) and vowels (Burnham and Torstensson, 1995). Burnham (Burnham et al., 1991; Burnham, 2003) showed an intensified reduction in perceptual discrimination of non-native speech contrasts at 6, but not 4 or 8, years. This is strongest in children with better reading and reading-related ability (Burnham, 2003; Horlyck et al., 2012), and is a function of duration of school experience, rather than maturation *per se* (Horlyck et al., 2012). Burnham suggests that this reduced attention to non-native speech sounds is a response to the onset of reading instruction and that it may assist reading processes, specifically phoneme-to-grapheme mapping. This is consistent with the fact that the reduced attention to non-native speech sounds is ameliorated by 8 years, an age at which reading usually becomes more automatic (Burnham, 2003).

No studies have yet investigated whether this second period of attunement also occurs for lexical tones. To that end, this study will test primary (elementary) school children in First Grade (6–7 years old) and Third Grade (8–9 years old) for their perception of non-native lexical tones.

### Tone perception and training—auditory and auditory-visual information

#### Auditory training of tone perception

Auditory training improves non-native tone perception in adult populations. For example, Wang et al. (1999) tested American-English adults in an auditory training regime for Mandarin tones involving learning the six possible pairings of the four Mandarin tones in eight sessions spaced over 2 weeks. There was a 21% increase in identification accuracy from pre-training to post-training test, an improvement which generalized to the perception of new stimuli by new speakers and which was maintained at 25% in a 6-month retention test (Wang et al., 1999). One of the strengths of the training was thought to be the use of a variety of monosyllabic Mandarin words and a variety of speakers. These will be implemented in the current study as well.

In another study, tone language Mandarin Chinese and non-tonal English speakers were trained to perceive Cantonese Chinese lexical tones (Francis et al., 2008). Both groups showed a similar initial performance and significant improvement in identification following training. However, English and Mandarin Chinese participants found particular tones difficult and others easier to identify, with English listeners improving significantly on the low-rising (23) and low–level (22) tones while Mandarin listeners showed significant improvement only on the low-falling (21) tone (Francis et al., 2008). While these results auger well for training non-tone language speakers to learn to perceive lexical tones, other studies have not been as successful with non-tonal speakers. Wayland and Guion (2003) investigated native English and native Chinese speakers' identification and discrimination of Thai tones. A significantly greater improvement from pre-training to post-training test was observed in the native Chinese group than in the native English group whose performance even declined over time. However, English speakers with some experience with Thai showed greater improvement in the perception of Thai tones compared to English speakers with no experience. Therefore, it can be concluded that previous lexical tone experience in a tone system, be it either as an L1 or an L2, may transfer to the perception of tones in a different tone system at least for adult learners (Wayland and Guion, 2003, 2004). In the study reported here, such prior tone language experience was investigated in children with respect to transfer to learning tones in a new unfamiliar tone system.

#### Auditory-visual tone perception and training

It is now well established that speech perception is multimodal, particularly with respect to auditory and visual information and particularly for consonants and vowels (McGurk and McDonald, 1976; Campbell et al., 1998; Vatiktiotis-Bateson et al., 2000). Evidence for auditory-visual perception of tone has been later in emerging. In two preliminary studies, Burnham found native Cantonese adults' identification of native tones presented in a Visual-Only (VO) mode was significantly better than chance for tones in running speech (but not words in isolation), for tones on monophthongal (but not diphthongal) vowels, and for contour (but not level) tones (Burnham et al., 2001a). In addition, both non-native Thai listeners and non-tonal Australian English adults were shown to make use of (presumably language-general) visual information in their discrimination of Cantonese tones (Burnham et al., 2001b).

Further studies have shown ubiquitous augmentation of visual tone perception in auditory-visual over auditory-only presentations (Mixdorff et al., 2005a,b,c; Smith and Burnham, 2012; Burnham et al., 2014). For instance Burnham et al. (2014), investigating the perception of Thai tones in noise, found better tone perception in AV than AO conditions irrespective of language background: visual augmentation was equivalent in tone language (Thai, Cantonese, Mandarin), pitch-accent (Swedish), and non-tone language (English) adults. Interestingly, Burnham et al. (2014) also found that non-tone-language English adults were much better than tone language or pitch-accent language adults in perceiving tone in VO situations (see also Smith and Burnham, 2012), presumably because those with no tone language experience use all available (e.g., visual) information for perceiving tones, while tone and pitch-accent language adults are accustomed to relying upon the perceptually more salient auditory information for tone.

#### Auditory-visual speech perception in children

Auditory-visual speech perception, at least for consonants, is evident early in development, even in infancy (Rosenblum et al., 1997; Burnham and Dodd, 2004; Desjardins and Werker, 2004). For example, 4½-month-old infants perceive the McGurk effect – auditory [ba] dubbed onto visual [ga] as “da” or “tha”– significantly more often than as “ba” (Burnham and Dodd, 2004). Nevertheless, there is further development of auditory-visual speech perception across childhood. In the original McGurk effect report (McGurk and McDonald, 1976) adults reported the auditory-visual fusion more than did children of 7 to 8 and 3 to 5 years. Subsequent studies have shown this reduced visual influence over age to be robust. There is more use of visual information by adults than by 4–6-year-old children (Massaro, 1984; Massaro et al., 1986), and there is a monotonic increase in visual speech perception across childhood from 5-, 7-, 9-, and 11-year-olds to adults (Hockley and Polka, 1994). This developmental increase is possibly related to articulation experience. Desjardins et al. (1997) showed that preschool children who make substitution errors in articulation are less influenced by visual cues than are children who can correctly produce consonants. In addition, between 6 vs. 8 years, the same ages as those tested in the study to be reported here, there is a large increase in the incidence of the McGurk effect in English-language (but not Japanese) children (Sekiyama and Burnham, 2008) which appears to be related to the onset of reading instruction (Erdener and Burnham, 2013).

### Tone perception and training in children

On the basis of the above studies, we may expect some effect of visual information on speech perception in school-age children. However, while there is ample evidence for this with consonants, there are, as yet, no studies on children's auditory-visual perception of tone or of training tone perception in children. Nevertheless, there *are* studies on the auditory training of tone perception in children (Wang and Kuhl, 2003; Sereno and Maniwa, 2006; Sereno, 2017). Wang and Kuhl (2003) trained monolingual American English 6-, 10-, and 14-year-old children and young adults to perceive Mandarin tones. Music, pictures, and sound effects were presented in the training program to engage the children and they also received rewards during the training. Training included six sessions spaced over 2 weeks with six different speakers of Mandarin Chinese. Accuracy in perceiving the Mandarin tones significantly improved in all age groups, but much more markedly in the young adults. It was suggested that the factors influencing lower performance among younger participants may be cognitive maturity resulting in difficulty in completing tasks, as well as experience with language in general. This study showed that six training sessions were effective and sufficient to improve tone perception at least in the older participants. Only auditory-alone training was used; no facial speech information was presented. In the study presented here, both auditory-only and auditory-visual training will be included with the expectation that auditory-visual training could enhance children's learning of non-native tones.

### Lexical tone perception in bilinguals

A bilingual person is one who displays language abilities in two languages that they use frequently in many aspects of their daily lives (Grosjean, 2010). Bilingualism plays various roles in children's language development. One key advantage is heightened metalinguistic awareness which relates to understanding the elements that make up language including rules and patterns (Campbell and Sais, 1995; Jensen, 2008). While bilingual children may perform less well than their monolingual peers on linguistic tasks, they invariably do better on executive control tasks (Friesen and Bialystok, 2012). Whether this then results in better ability to learn lexical tones is unknown, as there is no information thus far on any bilingual advantage for children learning lexical tones. Most lexical tone studies have been conducted with monolingual populations, and have shown that tone language experience facilitates non-native tone perception. One exception is a study by Singh and Foong (2012) who investigated the age at which Chinese-English bilingual infants are able to recognize and distinguish between non-phonemic and phonemic pitch and lexical tone contrasts in each language. In a word matching task 11-month-old (but not at 7.5- or 9-month-old) Chinese-English bilingual infants correctly recognized words whether they were pitch-matched or pitch-mismatched in English, but only correctly recognized words when they were pitch(tone)-matched in Mandarin. Thus, the perceptual attunement found for lexical tones early in infancy around 4 months appears to develop further in tone/non-tone language bilinguals such that by 11 months there is selective attunement depending on the language context.

The study reported here is the first to focus on the intricacies of training non-native lexical tone perception to monolingual vs. bilingual children with or without tone language experience. Four groups of primary (elementary) school students in two age groups, First grade (6–7 year-olds) and Third grade (8–9 year-olds), were trained (using either Auditory-Only or Auditory-Visual stimuli) to perceive non-native, Mandarin, lexical tone contrasts. Two of the four groups were bilingual: one bilingual group with two non-tonal language backgrounds: English and Arabic (**Bi-Eng/Arabic**); the other bilingual group with one non-tone, English, and one tone, Thai, language background (**Bi-Eng/Thai**). In addition, there were two groups of monolingual children – one non-tonal, English (**Mono-Eng**) and one tonal (**Mono-Thai**).

## The experiment: training non-native listeners to perceive mandarin tones

In this study, children were trained to perceive the four Mandarin tones using Auditory-Only (AO) or Auditory-Visual (AV) computer-based 4-alternative forced choice-identification tasks across six training sessions.

Since tone language experience has been shown to facilitate lexical tone perception, it is expected that children with tone language experience would be better able to perceive foreign lexical tones, and those with a non-tone language background will have difficulty perceiving tones. Thus perception accuracy and improvement over training on Mandarin tones is expected to be better for children with tone language experience (Bi-Eng/Thai and Mono-Thai groups) than those without (Bi-Eng/Arabic and Mono-Eng groups).

Moreover, it is expected that bilinguals (Bi-Eng/Thai and Bi-Eng/Arabic) should show better performance than monolinguals (Mono-Thai and Mono-Eng) due to greater metalinguistic awareness that comes with the ability to attend to and transfer across languages.

In addition, as there has been found to be visual augmentation of auditory tone perception in adults, it is expected that groups of children given Auditory-Visual training will perform better than those given Auditory-Only training, although this is proposed tentatively, as visual perception of tone has not yet been studied in children.

Finally, while there is only two years between the two age groups here, 6 and 8 years, it is possible that the reduced ability to perceive non-native speech contrasts in the second period of perceptual attunement around reading onset may affect the younger, 6-year-old, more than the older 8-year-old children.

As there has been found to be a relation between children's speech perception and reading and reading-related abilities (Burnham, 2003; Horlyck et al., 2012) and between children's phonological and tonological awareness and their reading ability (Burnham et al., 2011), we also included tests of English language phonological awareness—phoneme deletion and word and non-word reading ability—for the three groups with English as one of their languages (Bi-Eng/Arabic, Bi-Eng/Thai, Mono-Eng). It was expected that there would be a stronger relationship between phonological awareness and tone perception for the 8-year-olds than the 6-year-olds (given that at 6 years there is an intensification of perceptual attunement; Burnham, 2003) and possibly a greater phonological awareness with tone perception relationship for bilingual than monolingual children due to the former's greater metalinguistic awareness (Campbell and Sais, 1995; Jensen, 2008).

## Methods

### Participants

A sample of 81 primary school students participated in this study. The children were either bilingual (Bi-Eng/Thai or Bi-Eng/Arabic) or monolingual (Mono-Thai or Mono-Eng) and they had either a non-tone (Bi-Eng/Arabic or Mono-Eng) or tone (Bi-Eng/Thai or Mono-Thai) language background. Within each language group, there were two age groups, First Grade 6 to 7 years [6yo] and Third Grade 8–9 years [8yo], and within each language × age sub-group children were randomly assigned to either an Auditory-Only (AO) or an Auditory-Visual (AV) training group, prior to and irrespective of their scores on the Pre-training tests.

All participants' parents reported their children had normal hearing in both ears. Numbers, ages and distribution of the participants in each of the four language groups are as follows:

- Bi-Eng/Arabic: 24 children (16 female, *M*_age_ = 8.07, *SD* = 1.15) – 12 6yo (11 female, *M*_age_ = 7.09, *SD* = 0.60; 6 in the AO Training, and 6 in the AV Training group); 12 8yo (5 female, *M*_age_ = 9.05, *SD* = 0.54; 6 AO Training, 6 AV Training).
- Bi-Eng/Thai: 24 children (15 female, Mage = 8.00, *SD* = 1.24) – 12 6yo (10 female, Mage = 6.94, *SD* = 0.52; 6 AO Training, 6 AV Training); 12 8yo (5 female, Mage = 9.07, *SD* = 0.71; 6 AO Training, 6 AV Training).
- Mono-Eng: 17 children (11 female, *M*_age_ = 7.67, *SD* = 1.30) – 8 6yo (6 female, *M*_age_ = 6.48, *SD* = 0.56; 4 AO Training, 4 AV Training; 9 8yo (5 female, Mage = 8.73, SD = 0.64; 5 AO Training, 4 AV Training.
- Mono-Thai: 16 children (12 female, Mage = 7.5, *SD* = 1.20) – 8 6yo (5 female, Mage = 6.5, *SD* = 0.53; 4 AO Training, 4 AV Training); 8 8yo (7 female, Mage = 8.5, *SD* = 0.53; 4 AO Training, 4 AV Training).

Children's parents or guardians gave informed consent to participate in the experiment. For the bilingual groups, the Bi-Eng/Arabic children were recruited from St. Charbel's College and Al Jabal Karm El Mohr community, and the Bi-Eng/Thai group from Buddharangsee Thai Community Language School, both in Sydney, Australia. All participants' parents or guardians received an AUD50 gift voucher as reimbursement for travel expenses. For the monolingual groups, the Mono-Eng participants were recruited from Deerfield Elementary School, in Lawrence, Kansas, USA, where participants' parents or guardians received USD60 as reimbursement. The Mono-Thai children were recruited from Wat Baan Maa School in Ayutthaya, Thailand. The participants' parents or guardians received a gift voucher worth THB500 as travel expense reimbursement. All children in the study received small gifts and a certificate of participation at the end of each session. The criteria for bilingualism in this study for the two bilingual groups were (a) at least one parent was a native speaker of Thai or Arabic, depending on the group, and spoke to their child in that language on a daily basis; (b) the children systematically learned both languages either at their normal school or at a language school; and (c) the parents reported that their children used each language on a daily basis. Parents or guardians also filled out a questionnaire regarding their child's language and musical training background. Only one child in the Bi-Eng/Arabic and three in the mono-Eng groups had received musical training while about 50% of the Bi-Eng/Thai group were engaged in Thai musical training at the time of testing as a requirement of the Thai language Saturday school curriculum. The study was conducted under the Western Sydney University and the University of Kansas Human Research Ethics Committees' approval in accordance with the Code of Ethics of the World Medical Association (Declaration of Helsinki).

### Design

The study employed a mixed, 2 × 2 × 2 × 2 × (2 × 2) factorial design: 2 Language Background groups (bilingual or monolingual) and 2 Tone Language Experience groups (tonal or non-tonal) × 2 Age groups (6yo or 8yo) × 2 Training Modes (Auditory-Only [AO] or Auditory-Visual [AV]) between-subject factors × 2 Test Types (auditory-only [ao] or auditory-visual [av]) × 2 Test Phases (Pre-/Post-training) within-subject factors. The main dependent variable for accuracy was the percentage of correct tones identified. Participants in each Language × Age group were randomly assigned to either the AO or AV Training in equal numbers for each group. The order of the Test trials (i.e., ao and av) was counterbalanced between participants within groups. In addition, the three of the four groups who had English as (one of) their language(s) were also given phonological awareness tests, including a phoneme deletion and a reading ability (words and non-words) test.

### Perception test stimuli

Stimuli used in the experiment were auditory-visual recordings of monosyllabic Mandarin syllables from six native speakers of Beijing Mandarin Chinese (4 females and 2 males). One female speaker (Speaker F3) provided stimulus items for the introductory session and the practice test. Another female speaker (Speaker F4) provided stimulus items for the Pre- and Post-training Test Phase stimuli. Four other speakers, two female (F1, F2) and two male (M1, M2) provided stimulus items for the training sessions.

The recordings were conducted in a soundproof booth in the Face and Voice Lab at MARCS Institute for Brain, Behavior and Development, Western Sydney University. The speakers were asked to produce target syllables via an AKG lapel microphone connected to an SONY HD video camera. The recording sessions were recorded via Adobe Premiere Pro CS5 program (www.adobe.com). Sound was sampled at a frequency of 48,000 Hz. Target syllables were presented stationary in the center of a computer screen one at a time. Two separate files were then created from the same recording session for each stimulus, one for the Auditory-Only (AO) training and another for the Auditory-Visual (AV) training condition. Note that for the AO, a still image of the speaker was presented.

A total of 12 stimuli were created for the introductory session. These comprised Speaker F3 productions of 3 syllables (/ma/, /ni/, /ga/) with the four Mandarin tones (T55, T35, T214 and T51). Another set of 24 stimuli were created for the practice session. These comprised Speaker F3 productions of 3 syllables (/ma/, /na/, /p^*h*^a/), spoken with each of the four Mandarin tones, as well as one repetition of the resultant 12 trials.

A total of 96 stimuli (all words), 24 for each of the four Mandarin tones were created for the pre-training and post-training tests. These comprised speaker F4 producing 24 syllables on each of the four Mandarin tones. The stimuli were identical in the pre-training and post-training tests but were re-randomized in each test. These 24 syllables were [Pinyin: can, chu, chuang, di, fa, gu, guo, han, hou, lang, nao, pai, peng, qian, qiao, qie, qu, shao, tui, xiang, xing, xue, yu, zuo].

A total of 144 stimuli (words and non-words), not present in the pre-training and post-training tests or practice were created for the six training sessions (See Table A in the Supplementary Material for all training stimuli). For each session, 24 stimuli were presented by each of the four speakers (F1, F2, M1, and M2). Thus each training session included 96 trials, and stimuli were randomized in each training session.

### Phonological awareness tests

Three phonological awareness tests were used. A Phoneme Deletion test was adapted from Tyler and Burnham (2006) and McDougall et al. (1994) and consisted of three practice trials and 18 test trials. Children were asked to pronounce a word omitting a particular sound, e.g., “say “train”without the “t” sound.” Each correct response scored 1 and the dependent variable was the proportion of correct phoneme deletions. The task was presented via audio files in Microsoft PowerPoint on the laptops used for the perception test. The second two tests were the “Sight Word Efficiency” test consisting of 108 words and the “Phonemic Decoding Efficiency” test consisting of 66 phonotactically legal (in English) non-words, both from the Test of Word Reading Efficiency-Second Edition (TOWRE-2, Form A). The child was first given eight practice words or non-words to read aloud. Then, in each test, they were asked to read aloud as many words as possible in 45 s. The dependent variable is the number of words or non-words read accurately in 45 s.

### Procedure

The perceptual identification tests were run on DmDx software (Forster and Forster, 2003) in three different locations with up to four laptops running simultaneously in each location: Bi-Eng/Arabic and Bi-Eng/Thai in Australia, Mono-Eng in the USA, and Mono-Thai in Thailand. All audio was played through high-quality headphones connected to USB audio capture sound cards, interfaced with the laptops. The sound level was initially set at 65 dB HL for each participant, but adjusted, as required, to a comfortable listening level after the practice trials. An identification task was used. On each trial a single word was played through the headphones and children were instructed to identify which tone was played by responding on a USB-connected button box with four colored buttons, red, orange, yellow and green representing the four Mandarin tones, red for Tone 55, orange for Tone 35, yellow for Tone 214, and green for Tone 51. Participants were instructed to pay attention to both the auditory and visual aspects of the presentations in all sessions but there were no specific instructions given about what specific cues to attend to. Information sheets, consent forms, and language questionnaires were distributed to parents or guardians of the children prior to the first session and were collected from parents at the first session.

Training in the AO and AV training mode groups was exactly the same except that in AV training words were presented auditory-visually via the articulating speaker's face, and in AO training words were presented auditorally with the dynamic video turned off and a static image of the speaker's face on the screen. The tone training program consisted of six sessions. The first was an introductory session to allow participants to become familiarized with the task, and included pre-training tests (both ao and av) for all participants irrespective of training (AO or AV) mode; a practice session (with AO or AV training depending on the training group): and the first training session (AO or AV). The second to fifth sessions consisted of only a training session (AO or AV, depending on group) and the sixth session consisted of AO or AV training plus post-training tests (ao and av tests for all participants irrespective of training group). Feedback (“Good Job!!” for correct responses; “Sorry!!” for incorrect responses, and “Sorry, Press Faster!!” for missing responses) was given in practice and in AO or AV training sessions but not in the pre-training or post-training ao and av tests. In all training sessions, participants were also rewarded with a short cartoon clip every time they made three consecutive correct responses. The six sessions were scheduled at two sessions per week over 3 weeks. In the third session, the three groups with English as one of their languages (Mono-Eng, Bi-Eng/Arabic, and Bi-Eng/Thai) also completed the three phonological awareness tasks, Phoneme Deletion, and Word reading and Non-word reading.

Testing and training were conducted in quiet classrooms at each school; or at MARCS BabyLab, Western Sydney University Bankstown Campus; or at public library near the participant's home. A maximum of four children were tested at one time, using four identical laptops and associated hardware and software.

## Results

The results are presented in three parts: (a) a comparison of total raw accuracy in pre-training vs. post-training tests, irrespective of Training Mode (AO/AV) and test mode (ao/av) in an Age (6 vs. 8 years) × Language Background (Bilingual vs. Monolingual) × Tone Language Experience (Tonal vs. Non-tonal) × (Mean Test Score – Pre-/Post-Training) design with repeated measures on Pre- vs. Post-Training scores; (b) an analysis of a percentage gain due to training dependent variable derived from the Pre- and Post-Test scores (see formula below) in an Age × Language Background × Tone Language Experience × Training Mode (AO/AV) × (ao vs. av Tests) design, with repeated measures on ao vs. av tests; and (c) a set of correlations between the phoneme deletion and word and non-word reading ability tests and with the pre- and post-tests and gain due to training for the three English-speaking groups (Mono-Eng, Bi-Eng/Arabic, Bi-Eng/Thai) for whom data on the phonological awareness and reading tests was collected.

### Raw accuracy

Raw percentage correct data were first analyzed to show the absolute level of performance Post-Training compared to Pre-Training as a product of the group factors. A 2 × 2 × 2 × (2) Analysis of Variance (ANOVA) was conducted with Age, Language Background and Tone Experience as between-subject factors and Phase (Pre- vs. Post-training test), as the within-subject factor. All factors have two levels so no planned contrasts were required. Alpha was set at 0.05 and the effect sizes are given for significant differences (critical *F* = 3.898).

**Figure 2:**
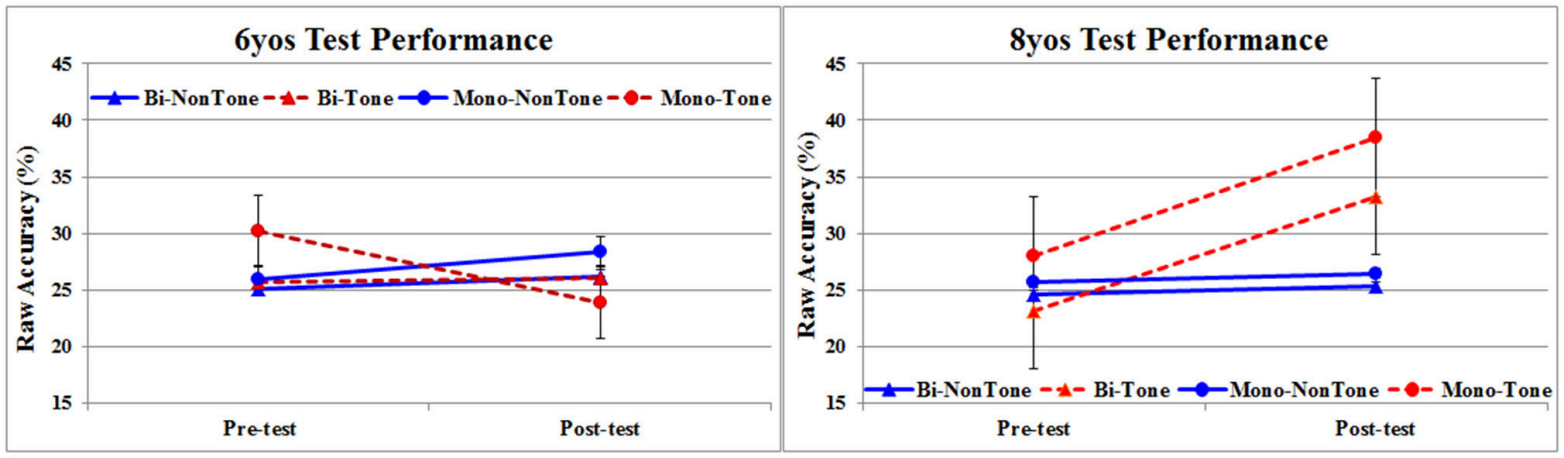
Mean percentage correct on Pre-training and Post-training test trials as a function of Age (6yo vs. 8yo), Language Background (Bilingual vs. Monolingual) and Tone Experience (Non-Tone vs. Tone). Error bars represent standard errors.

The results are graphically presented in Figure 2. As can be seen there was a general improvement from pre-training to post-training and this Phase main effect was significant, *F*_(1,65)_ = 7.61, *p* < 0.01, ηp^2^ = 0.077, with Post-Training Mean = 28.43, and *SD* = 0.09, and Pre-Training Mean = 25.76, and *SD* = 0.06.

As can be seen in Figure 2, there was Pre- to Post-Training improvement for three of the four 6-year-old groups, and all of the four 8-year-old groups, with other interactions also apparent. Accordingly, while the Phase main effect was unaffected by Language Background, Monolingual vs. Bilingual, it was qualified by Age and Tone Experience: there was a Phase × Age, *F*_(1,65)_ = 9.90, *p* < 0.005, ηp^2^ = 0.395, and a Phase × Age × Tone Experience, *F*(1,65) = 15.40, *p* < 0.001, ηp^2^ = 0.505, interaction. As can be seen in Figure 2, these interactions are due to (i) greater improvement from pre- to post-training by 8-year-olds than by 6-year-olds, and (ii) especially greater improvement for 8-year-olds with Tone Language experience, irrespective of whether the tonal experience is in a monolingual or bilingual context.

The *decrease* in performance from pre- to post-training by the monolingual tone language (Thai) 6-year-olds is puzzling. These children had just begun instruction in reading and writing at school, including learning the orthographic representation of Thai tones (a regular but complicated 4-way interaction of initial consonant class, final consonant manner, vowel length, and tone diacritics (Kasisopa et al., 2013, 2016; see Davis et al., 2015). It is possible that these, as yet non-automatic controlled, processes involved in learning the orthographic representation of Thai tones coupled with intensive training on foreign (Mandarin) tones, resulted in overload and confusion at the perceptual level interference from L1 phoneme-to-grapheme/grapheme-to-phoneme levels. This explanation is clearly speculative and requires further research.

### Performance gain

While the above analysis shows effects of training on tone perception, it may be noted that many of Pre- and some of the Post-test scores hover around chance level (25%, given there are 4 Mandarin tones). This raises the issue of the degree of improvement given the initial level of performance and the equivalence of improvements from an initial level of chance responding vs. a higher level of initial responding. To accommodate such differences a dependent variable was derived as follows:

$$\text{Performance Gain }=\left(({\text{post}}_{\text{test}}\%\text{ correct }-\;{\text{Pre}}_{\text{test}}\;\%\text{ correct}\right)/{\text{Pre}}_{\text{test}}\;\%\text{ correct}){)}^{*}100\%$$

**Figure 3:**
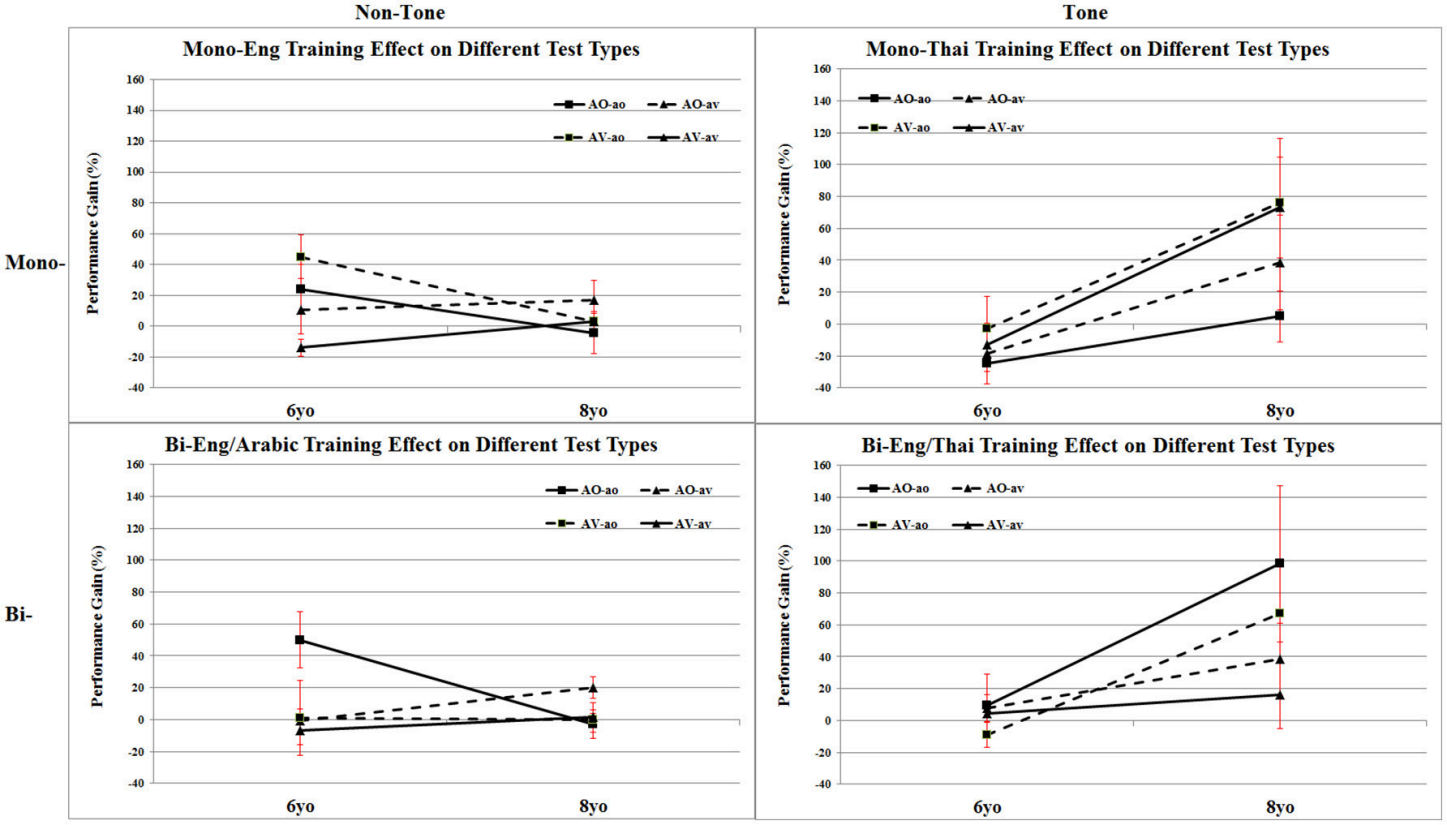
Mean percentage of Pre- to Post-training improvement gain by each of the four language groups at 6 and 8 years, with AO or AV Training ao and av Test Types. Error bars represent standard errors.

Thus if a child had 20% correct on Pre-Training and 30% on Post-Training—the Performance Gain would be 50%; or if there was the same absolute increase of 10% from 50% on Pre-training to 60% on Post-training, the percentage improvement would be 20%. Thus this measure takes into account the initial level of performance in the pre-training test and represents the percent improvement in relation to that level. Mean and Standard Error Performance Gain for each of the four Language Background × Bilingual Status groups are shown for AO/AV training groups in ao and av tests in Figure 3 as well as in Table 1 alongside the number and percentage of participants who showed pre- to post-training improvement in each group (see also Table B in the Supplementary Material for individual Performance Gain scores for each participant).

**Table 1:** Mean (and *SD*) Performance Gain and number and percent of participants who improved in each group.

		**Post-Pre**	**No. improved**	***n***	**% improved**	**Post-Pre**	**No. improved**	***n***	**% improved**
**Mono-Eng**		**6 year-old (*****N*** = **8 Participants)**				**8 year-old (*****N*** = **9 Participants)**			
AO Train	ao Test	23.98 (32.41)	3	4	75	−4.56 (29.27)	1	5	20
	av Test	10.54 (31.59)	2	4	50	16.70 (29.32)	3	5	60
AV Train	ao Test	44.93 (28.46)	4	4	100	3.20 (11.91)	1	4	25
	av Test	−13.99 (10.66)	0	4	0	3.18 (12.88)	2	4	50
**Mono-Thai**		**6 year-old (*****N*** = **8 Participants)**				**8 year-old (N** = **8 Participants)**			
AO Train	ao Test	−24.58 (10.83)	0	4	0	4.80 (31.91)	3	4	75
	av Test	−18.51 (38.18)	3	4	75	38.60 (59.69)	3	4	75
AV Train	ao Test	−2.94 (40.11)	2	4	50	76.31 (80.27)	3	4	75
	av Test	−13.02 (20.71)	1	4	25	73.05 (63.40)	4	4	100
**Bi-Eng/Arabic**		**6 year-old (N** = **12 Participants)**				**8 year-old (N** = **12 Participants)**			
AO Train	ao Test	49.98 (43.26)	6	6	100	−2.83 (22.18)	3	6	50
	av Test	−0.71 (17.45)	3	6	50	20.15 (16.12)	5	6	83
AV Train	ao Test	1.08 (58.19)	2	6	33	0.21 (8.43)	3	6	50
	av Test	−6.69 (22.81)	2	6	33	1.46 (22.92)	3	6	50
**Bi-Eng/Thai**		**6 year-old (N** = **12 Participants)**				**8 year-old (N** = **12 Participants)**			
AO Train	ao Test	9.47 (47.88)	2	6	33	98.24 (120.36)	6	6	100
	av Test	7.77 (21.00)	3	6	50	38.31 (56.05)	4	6	67
AV Train	ao Test	−8.97 (19.15)	2	6	33	67.00 (75.50)	5	6	83
	av Test	4.65 (7.88)	4	6	67	16.38 (52.05)	2	6	33

**Figure 4:**
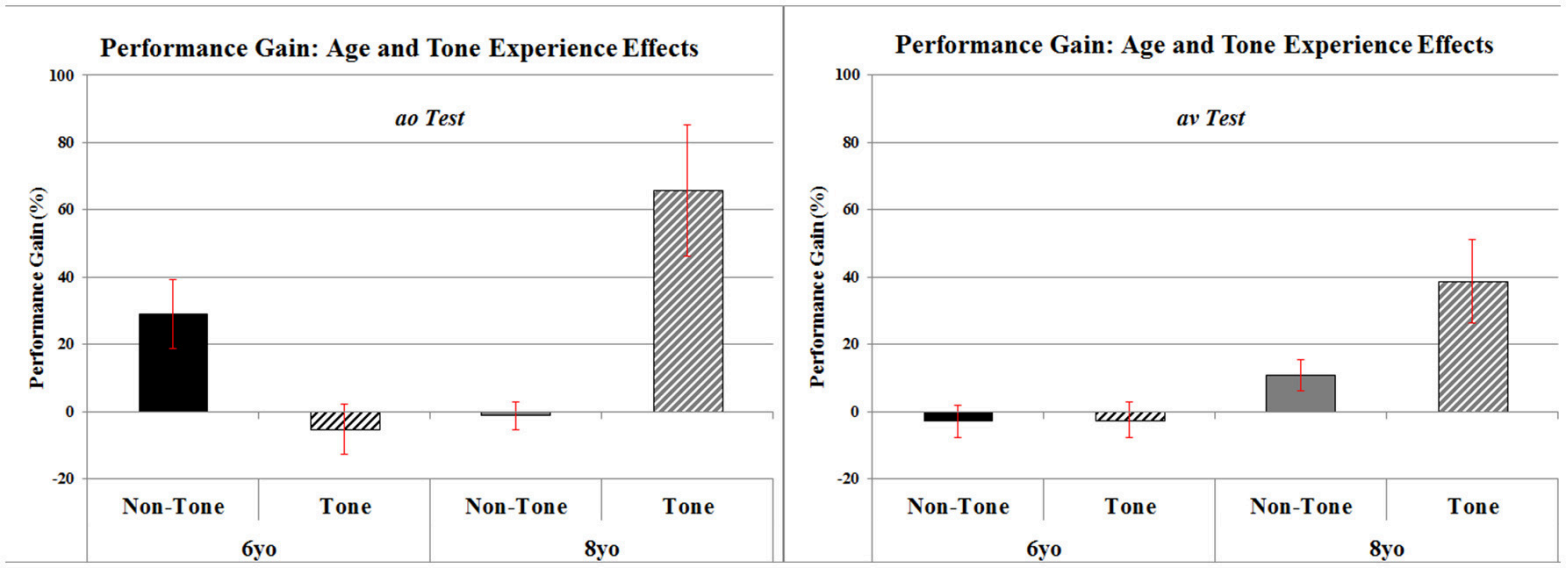
Mean percentage of Pre- to Post-training performance gain for Mandarin Tone Perception including effects of Age (6yo vs. 8yo), Tone Experience (Non-Tone vs. Tone), and Test Types (ao vs. av). Error bars represent standard errors.

Performance Gain scores were analyzed in an Age × Language Background x Tone Experience × Training Mode between-subject factor × Test Type (ao or av) within-subject factor ANOVA. The only significant main effect was for Age, *F*_(1,65)_ = 8.09, *p* < 0.01, ηp^2^ = 0.111. Age also interacted with two other factors: there was an interaction of Age × Tone, *F*_(1,65)_ = 15.19, *p* < 0.001, ηp^2^ = 0.189, and of Age × Tone × ao/av test, *F*_(1,65)_ = 9.54, *p* < 0.01, ηp^2^ = 0.128. This set of results is represented in Figure 4. As can be seen, 8-year-olds showed more Performance Gain than 6 year-olds. There was more Performance Gain for Tone language than Non-Tone language background children, but this was only evident in the 8-year-olds. Finally, while the Tone > Non-Tone advantage for 8-year-olds was evident in both ao and av tests, Performance Gain was greater when indexed in ao tests.

**Figure 5:**
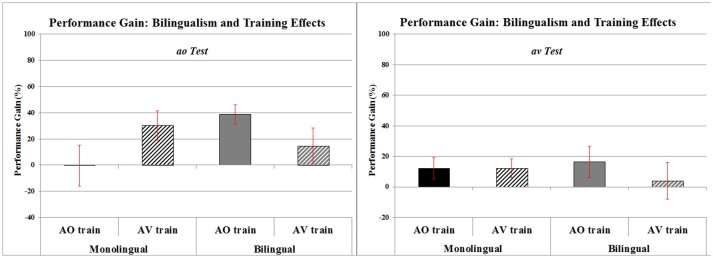
Mean percentage of Pre- to Post-training performance gain for Mandarin Tone Perception including effects of Bilingualism (Mono- vs. Bi-), Training (AO vs. AV) and Test Types (ao vs. av). Error bars represent standard errors.

The above Age and Tone Language background results are independent of whether the children were monolingual or bilingual and whether they were trained with AO or with AV stimuli. Turning to Training Mode and Monolingual/Bilingual, the Training Mode and Monolingual/Bilingual interaction, and the Training Mode × Monolingual/Bilingual × ao/av interaction were both very close to significance Training Mode × Monolingual/Bilingual, *F*_(1, 65)_ = 3.91, *p* > 0.05, ηp^2^ = 0.057; Training Mode × Monolingual/Bilingual × ao/av tests, F = 3.76, p>0.05, ηp^2^ = 0.055 (critical *F* = 3.98). Given these close to significant interactions and the significant interaction of ao vs. av tests with Age and Tone Language results above, and in order to avoid a Type II error in this first test of the effect of training mode on lexical tone perception, these two approaching significance results were followed up in simple effect tests of Training Mode × Monolingual/Bilingual at each level of the test type, ao tests and av tests. These revealed a non-significant Training Mode × Monolingual/Bilingual interaction for av tests, *F*_(1, 65)_ = 0.70, *p* > 0.1, ηp^2^ = 0.011, but a significant Monolingual/Bilingual interaction for ao tests, *F*_(1, 65)_ = 5.19, *p* < 0.03, ηp^2^ = 0.074. This set of results is represented in Figure 5. As can be seen Bilingual participants show greater Performance Gain after training with AO stimuli, whereas Monolingual participants show greater Performance Gain after training with AV stimuli, and this is especially the case when indexed by ao test trials.

### Correlations with phonological awareness and reading

Correlations, with age partialed out, between the phoneme deletion, word and non-word reading tests with the pre-training, post-training, and performance gain due to training were conducted for the three groups with English as one of their languages (Mono-Eng, Bi-Eng/Arabic, Bi-Eng/Thai).

There were, not surprisingly, correlations between the three language measures –phoneme deletion and word reading *r*_(62)_ = 0.50, *p* < 0.001, phoneme deletion and non-word reading *r*(62) = 0.59, *p* < 0.001, and word and non-word reading, *r*_(62)_ = 0.75, *p* < 0.001.

More important are correlations between any of the three language measures and the tone training scores. The only significant correlation of this nature was between phoneme deletion and the pre-training av-test, *r*_(62)_ = 0.26, *p* < 0.05. This indicates that children's phonological awareness, in this case their proficiency on a phoneme deletion task, is positively related to their initial identification of the four Mandarin tones presented in auditory-visual mode.

## Discussion

### Summary of results

This study examined the role of tone vs. non-tone language experience, monolingualism vs. bilingualism, and auditory-only vs. auditory-visual training of foreign lexical tone contrasts. The results are summarized under four headings below, followed by discussion of the results.

#### Training—effects of age and language factors

Training was effective: there was a general improvement in performance from pre- to post-training. Training was most effective for 8-year-olds; 6-year-olds showed only limited effects of training. Training was more effective if children had tone language experience, an advantage evident in the 8- but not the 6-year-olds. These effects of age and tone language on training were most clearly indexed by the ao rather than the av pre- and post-training tests.

#### Language background and training mode

There was a differential effect for the type of training: Monolingual children improved markedly with AV training but not at all with AO training, whereas Bilingual children improved markedly with AO training and to a lesser extent with AV training. However, these effects were only apparent when indexed by ao tests.

#### Correlation with language measures

For children with English as their only, or as one of their, language(s), proficiency on a phoneme deletion task was positively related to Mandarin tone identification in auditory-visual pre-training test trials. As this was before training began, it shows that those children good at manipulating phonemes in (one of) their native language(s) were also good at perceiving what were completely novel phonological elements for the Mono-Eng, the Bi-Eng/Arabic and the Bi-Eng/Thai groups. This advantage did not extend to training, there was no advantage for good phoneme deleters in learning about foreign tones, just in their initial perception of foreign tones.

The results bear on a number of issues which are discussed below ahead of a discussion of limitations and suggestions for future research.

##### Age

There are two possible reasons why training was more effective with the older 8-year-olds than the younger 6-year-olds: task difficulty, and reduced sensitivity to foreign sounds. First it may be that the task employed here was demanding in terms of the degree of sustained attention required. For example, while in the procedure used here the pre- and post-training trials were the same, the training trials incorporated variation of both speakers and words. Wang et al. (1999) trained adults on a variety of monosyllabic Mandarin words spoken by a variety of speakers and found especially resilient learning. In an adaptation for children Wang and Kuhl (2003) also found a high degree of learning. However, over their six training sessions they graded the difficulty of the tasks (2 weeks ABX, 2 weeks 2AFC identification, then 2AFC with speaker variation) and within each pair of sessions they trained easier tone pairs first. While the Wang and Kuhl (2003) study and the study reported here shared the variability of speakers and words, here the task would have been more difficult because (i) tasks were not graded and (ii) a single presentation 4AFC identification task was used. It remains for future studies to adapt the procedures here and those in the Wang studies (Wang et al., 1999; Wang and Kuhl, 2003) to derive optimal, L2 training regimes especially for younger, e.g., 6-year-old children.

Secondly, irrespective of task difficulty, 6-year-old children may have reduced sensitivity to foreign sounds. Burnham and colleagues (Burnham et al., 1991; Burnham, 2003) investigating what has been called a second period of perceptual attunement, have shown that 6-year-olds, compared with both 8-year-olds and also 4-year-olds, have reduced sensitivity to L2 sounds and suggest that this is an adaptive device which facilitates attention to the difficult task of phoneme-to-grapheme mapping involved in reading. Burnham contends that at 4 years this process has not begun, and by 8 years the process has become relatively automated, whereas at 6 years this attentional filtering is most useful. Whether this explains the results here cannot be fully ascertained without a 4-year-old comparison group, and it remains for future research to investigate this issue further.

##### Test trials and generalization of training

In this experiment children were given both ao- and av-test trials pre- and then post-training. The training was either with AO stimuli in one group and AV stimuli in another group. In addition, the stimulus words and speakers were different in the pre- and post-training test phase on the one hand and in the Training trials on the other. Therefore, generalization of training can be indexed in two ways. First, any improvement after training, can be considered generalization because the training and test stimuli differed (although there could be an across-the-board improvement because the pre- and post-training stimuli were from the same pool). In this sense then, any performance gain from pre- to post-training, such as those gains found in this study, can be considered as both learning, and generalization of learning. Second, generalization can be indexed by any performance gain across both the ao- and the av-*tests*, irrespective of whether the *training* used AO or AV materials. A confounding factor in the interpretation of the results with respect to this type of generalization is that ao-tests proved to be more sensitive indices of performance gain than were av-tests. Nevertheless, it can be concluded that, in general, generalization of training was best for 8-year-olds, and especially for 8-year-olds with tone language experience whether that be monolingual (Mono-Thai) or bilingual experience (Bi-Eng/Thai).

##### Tone language experience

Participants with tone-language experience (the Bi-Eng/Thai and Mono-Thai groups) benefitted more from training than those with no tone language experience (Bi-Eng/Arabic and Mono-Eng), irrespective of whether the children were monolingual or bilingual. In addition, those with tone language experience (especially the 8-year-olds) also showed better generalization of training across test type—ao and av. This supports previous findings that tone language experience facilitates adult lexical tone perception (e.g., Burnham et al., 2014) and extends these findings to children. Moreover, these data provide information about two aspects of language learning. First, the data tell us that there is some perceptual or conceptual information about lexical tones that is general across tone languages (or at least for the two tone languages here, Mandarin (the target language) and Thai (the language experience language). Second, the data tell us that any metalinguistic advantage or extra skills learned as a product of learning more than one language is independent of the skills required for learning to perceive lexical tone in a tone language. Each of these is discussed in further detail below.

##### Task difficulty and differences between tone languages

Mandarin and Thai tone inventories differ on a number dimensions: Mandarin has 4 tones and Thai 5; Thai has 2 level tones and 3 contour tones, Mandarin has 1 level and 3 contour tones; all 5 Thai tones are of similar duration, whereas Mandarin tones differ markedly in duration. Thus Mandarin and Thai are quite distinct with respect to their tones and this has two interesting implications with respect to the results obtained here. Firstly, given these differences, it is reassuring that there was an effect of (Thai) tone language background on the learning of the target tones in Mandarin, i.e., that there was transfer of learning from Thai tones to learning Mandarin tones. Second, the differences between Thai and Mandarin may have played a part in the relatively small performance gains in tone perception here. Further studies in which the background language and target tone language are more similar with respect to their tones, e.g., Thai and Cantonese (6 tones: 3 level and 3 contour, all tones of similar duration), may result in more performance gains. More generally, the relative salience of differences between tones within a particular language and the relative difficulty of discriminating tones in one tone system vs. that in another system is largely unknown (but see Burnham et al., 2017). Recent studies have shown that tone perception develops for a specific tone system (Yeung, et al., 2013) and that non-native tone language speakers have difficulty with tones that are similar or overlap with their native tone systems (Hao, 2012). Much more research is required on what particular parameters make particular tones or tone systems easier or more difficult to learn.

##### Monolingual and bilingual children

While monolingual vs. bilingual status of the children did not in itself facilitate tone learning in children, it did contribute to the mode of training that was most effective, as measured by the performance gain between pre- and post-training ao-test trials. The Auditory-Visual (AV) mode of training was the most effective for monolingual children, whereas for bilingual children Auditory-Only (AO) training, and, to a lesser extent, AV training resulted in performance gains. The source of this difference is not clear. One possibility is that exposure to a greater range of linguistic differences and devices, as would be the case for bilingual children, allowed them to (i) learn from a range of parameters, including auditory information alone or auditory and visual information combined, and (ii) learn that, even though there is visual information for tone (Burnham et al., 2001a,b, 2014; Smith and Burnham, 2012) the auditory information is by far the most salient. This is speculative and requires more definitive evidence.

##### Phonological awareness

English phoneme deletion ability (in the Mono-Eng, Bi-Eng/Arabic, and Bi-Eng/Thai groups) was positively related to pre-training auditory-visual test trial performance. Although there was no relationship here between reading and tone perception, the results are reminiscent of those of Burnham et al. (2011) who found a significant relationship between Thai children's reading ability and their phonological and tonological awareness, and between Australian English children's reading and their phonological awareness. Thus here, the ability to manipulate phonemes is related to the ability to perceive foreign speech sounds and in Burnham et al. (2011) reading ability is related to the ability to manipulate (foreign) phonemes and tonemes. Further research is required to investigate the nature of any three-way relationship between reading, phonological awareness, and foreign speech sound (and of especial interest here, lexical tones), the findings of which could be relevant to children's propensity to learn a second language, especially a tone language.

### Limitations and future directions

A number of limitations can be noted.

#### Training and test

The post-training test implicitly tested for generalization across speakers and words, and, in addition, these trials provided implicit tests of generalization from training mode (be it AO or AV) to test mode, as both ao and av tests were given irrespective of training. The downside of this is that tests between trained and untrained stimuli and voices could not be conducted. It is possible that children, even the younger 6-year-olds, may have performed better on trained than untrained stimuli and voices. This should be remedied in future studies. The upside is that any improvement as a result of training indicated generalization of training. So the performance gains obtained here, while modest, are robust.

A related point concerns *variability*. As discussed above, variability improves the robustness of learned distinctions (Wang et al., 1999), but variability should be optimized for the age and maybe the language background of the children. Here it was not.

A final point on this theme is that for both clusters of results—the age and tone language experience cluster, and the training mode and monolingual/bilingual language experience cluster—the ao-tests were more sensitive measures of improvement than were the av-tests. And, even though auditory and auditory-visual modes differentially affected training outcomes in monolingual and bilingual children, the indexation of such training was still generally better on ao-tests. The reason for this is unclear. In future studies it would seem that ao-tests should be preferred.

#### Phonological awareness

We included English language tests of phonological awareness here and found a positive relationship between phoneme deletion and pre-training auditory-visual test performance. Future studies should investigate this further by including reading tests across languages, phonological awareness tests across languages, and also tests of morphological awareness (McBride-Chang et al., 2003) and even executive function, in order to determine predictors of good lexical tone learning.

#### Instructions

No specific instructions were given. Children were simply told to pay attention to both the auditory and visual aspects of the speakers as we wished to determine whether children naturally pick up relevant lexical tone cues in an experimental setting. In real-life L2 learning situations such experimentally objective procedures may not be desired; indeed any relevant cue could and should be made available. In this regard, Chen and Massaro (2008) tested Mandarin perceivers' Visual-Only (VO) identification of the four Mandarin tones. (Remember that Mandarin language adults are worse than English language adults in VO tone perception—Smith and Burnham, 2012; Burnham et al., 2014). Initially the Chen and Massaro adults performed only just above chance and were better for the 55 and 214 tones than the 35 or the 51 tones. In a follow-up test adults were told about visible movements in tone perception and instructed to pay attention to movements of the neck, head, and mouth. Visual-Only tone perception improved significantly. Further work on perceivable visible cues for tone perception is required to facilitate L2 tone learning regimes.

#### Tone difficulty

The Chen and Massaro (2008) results also raise the issue of the relative difficulty of identification of individual tones and discrimination of tone pairs. Although the results of this study reported here were based on perception across all Mandarin tones, the data also showed some differences of how the participants of different ages and language backgrounds learned the Mandarin tones in this study. Details of performance on the different tones for each language background group and each age are shown in Table C (Supplementary Material) and some comments on these are provided here. Generally, high Static tone (T55) was the easiest tone to learn for monolingual non-tonal group while the Dynamic tones (either T241 or T51) were the most difficult. The results for the monolingual tonal group were exactly the opposite: the Static (T55) the most difficult to learn while the dynamic tones (T214 and T55) were the easiest. The data is a little less definite for the bilingual language background groups. Nevertheless, it appears that 6 year-olds in both bilingual groups found the Static tone (T55) the easiest to learn while the other Dynamic tones (T35, T241, and T51) were similarly difficult; whereas the 8 year-old bilingual groups found that the Dynamic (T214) was the easiest to learn. The fact that the participants WITH A TONAL BACKGROUND found the generally difficult DYNAMIC tones T35 and T214 (Chang, 2011) in Mandarin relatively easy to learn in this study is quite interesting. However, as the task used in this study was tone identification, some distinctive contours, rising and dipping, of these two rising tones might help in identifying them. The results might well be different if participants were asked to discriminate between these two rising tones; the task might be much more difficult. Future work must take into account such differences, but at the moment there are no objective criteria for determining difficulty of tone perception within and between languages. We (Burnham et al., 2017) are currently collecting data on the perception of tone pairs from three different tone languages by adults from five different language backgrounds in order to leach out some such criteria.

## Conclusions

The results of this study show that 8-year-olds and to some extent 6-year-olds can learn to identify Mandarin tones. The training procedure included feedback during practice and training sessions and rewards in training of animated cartoons after three consecutive correct responses. The same stimuli were used in pre- and post-training, but there was considerable variability of training stimuli in terms of speakers and the word contexts, variability that perhaps hindered learning in children of 8- and especially 6-years of age.

Nevertheless, there was successful training of 6- and 8-year-olds to identify non-native lexical tones. The main findings were that tone language experience with Thai facilitated learning to identify Mandarin tones, irrespective of whether the experience was monolingual Thai, or bilingual Thai-English. Monolingual vs. bilingual experience *per se* had no effect on tone training. However, the modality of training appeared to be differentially effective: as indexed in ao-test trials, monolingual children improved markedly with Auditory-Visual training stimuli but not at all with Auditory-Only training, whereas bilingual children improved markedly with Auditory-Only training and to a lesser extent with Auditory-Visual training. These results suggest an interesting interaction between language experience and training method that may have important implications for L2 training techniques and facilitate L2 training regimes.

## Author contributions

BK and LE-KA experimental design, data collection and analyses, data management, manuscript drafting and revising, approving the manuscript to be published and Agreeing to be accountable for all aspects of the work in this manuscript. AJ, JAS, and DB experimental design, supervision of data collection and data management, manuscripting drafting and revising, approving the manuscript to be published, and agreeing to be accountable for all aspects of the work in this manuscript.

### Conflict of interest statement

The authors declare that the research was conducted in the absence of any commercial or financial relationships that could be construed as a potential conflict of interest.

## Footnotes

^1^These numbers are Chao tone values which indicate tone height and contour at the start, (middle), and end of the syllable (Chao, 1930).

## References

[B1] Abramson A. S. (1978). Static and dynamic acoustic cues in distinctive tones. Lang. Speech 21, 319–325. 10.1177/002383097802100406 750791

[B2] Burnham D. (2003). Language specific speech perception and the onset of reading. Read. Writ. Interdiscip. J. 16, 573–609. 10.1023/A:1025593911070

[B3] Burnham D. Ciocca V. Stokes S. (2001a). Auditory–visual perception of lexical tone, in Proceedings of the 7th Conference on Speech Communication and Technology, eds Dalsgaard P. Lindberg B. Benner H. Tan Z. H. (Scandinavia: EUROSPEECH 2001), 395–398. Available online at: http://www.isca-speech.org/archive/eurospeech_2001

[B4] Burnham D. Dodd B. (2004). Auditory–visual speech integration by pre-linguistic infants: perception of an emergent consonant in the McGurk effect. Dev. Psychobiol. 44, 204–220. 10.1002/dev.20032 15549685

[B5] Burnham D. Kasisopa B. Reid A. Luksaneeyanawin S. Lacerda F. Attina V. (2014). Universality and language-specific experience in the perception of lexical tone and pitch. Appl. Psycholinguist. 77, 571–591. 10.1017/S0142716414000496

[B6] Burnham D. Kim J. Davis C. Ciocca V. Schoknecht C. Luksaneeyanawin S. (2011). Are tones phones? J. Exp. Child Psychol. 108, 693–612. 10.1016/j.jecp.2010.07.008 21087775

[B7] Burnham D. Lau S. Tam H. Schoknecht C. (2001b). Visual discrimination of Cantonese tone by tonal but non-Cantonese speakers, and by non-tonal language speakers, in Proceedings of Auditory–Visual Speech Perception Conference 2001 (AVSP 2001), eds Massaro D. Light J. Geraci K. (Adelaide, SA: Causal Productions), 155–160.

[B8] Burnham D. Mattock K. (2010). Auditory Development, in Handbook of Infant Development, Vol. 1 Basic Research 2nd Edn, eds Bremner G. Wachs T.D (Chichester: Wiley-Blackwell), 81–119.

[B9] Burnham D. Singh L. Kasisopa B. Wong P. Fu C. Wewalaarachchi D. (2017). The tone atlas, step1: perceptual salience of Thai, Cantonese, Beijing and Singaporean Mandarin Tones for Thai Adults, in Proceedings of the 11^th^ International Seminar on Speech Production (ISSP 2017), (Tianjin),141–144.

[B10] Burnham D. K. Earnshaw L. J. Clark J. E. (1991). Development of categorical identification of native and non-native bilabial stops: infants, children and adults. J. Child Lang. 18, 231–260. 10.1017/S0305000900011041 1874826

[B11] Burnham D. K. Torstensson C. (1995). The development of phonological bias: perception and production of Swedish vowels tones by English speakers, in Proceedings of the International Congress of Phonetic Sciences, eds Elenius K. Branderud P. (Stockholm: KTH and Stockholm University), 4, 558–561.

[B12] Campbell R. Dodd B. Burnham D. (1998). Hearing by Eye II: Advances in the Psychology of Speech Reading and Auditory–Visual Speech. East Sussex: Psychology Press.

[B13] Campbell R. Sais E. (1995). Accelerated metalinguistic (phonological) awareness in bilingual children. Br. J. Dev. Psychol. 13, 61–68. 10.1111/j.2044-835X.1995.tb00664.x

[B14] Chang Y. S. (2011). Distinction between mandarin tones 2 and 3 for L1 and L2 Listeners, in Proceedings of the 23rd North American Conference on Chinese Linguistics (NACCL-23), 1, 84–96.

[B15] Chao Y. R. (1930). A system of tone-letters. Le Maitre Phonetique 45, 24–27.

[B16] Chen T. H. Massaro D. W. (2008). Seeing pitch: visual information for lexical tones of Mandarin-Chinese. J. Acoust. Soc. Am. 123, 2356–2366. 10.1121/1.2839004 18397038 PMC2811545

[B17] Choi J. Cutler A. Broersma M. (2017). Early development of abstract language knowledge: evidence from perception-production transfer of birth-language memory. R. Soc. Open Sci. 4:160660. 10.1098/rsos.160660 28280567 PMC5319333

[B18] Davis C. Schoknecht C. Kim J. Burnham D. (2015). The time course for processing vowels and lexical tones: reading aloud Thai words. Lang. Speech 59(Pt. 2), 196–218. 10.1177/0023830915586033 27363253

[B19] Desjardins R. N. Rogers J. Werker J. F. (1997). An exploration of why preschoolers perform differently than do adults in audiovisual speech perception tasks. J. Exp. Child Psychol. 66, 85–110. 10.1006/jecp.1997.2379 9226935

[B20] Desjardins R. N. Werker J. F. (2004). Is the integration of heard and seen speech mandatory for infants? Dev. Psychobiol. 45, 187–203. 10.1002/dev.20033 15549681

[B21] Erdener D. Burnham D. (2013). The relationship between auditory–visual speech perception and language-specific speech perception at the onset of reading instruction in English-speaking children. J. Exp. Child Psychol. 116, 120–138. 10.1016/j.jecp.2013.03.003 23773915

[B22] Forster K. I. Forster J. C. (2003). DMDX: a windows display program with millisecond accuracy. Behav. Res. Methods Instrum. Comput. 35, 116–124. 10.3758/BF03195503 12723786

[B23] Francis A. Ciocca V. Ma L. Fenn K. (2008). Perceptual learning of Cantonese lexical tones by tone and non-tone language speakers. J. Phon. 36, 268–294. 10.1016/j.wocn.2007.06.005

[B24] Friesen D. C. Bialystok E. (2012). Metalinguistic ability in bilingual children: the role of executive control. Riv. Psicolinguist. Appl. 12, 47–56. 24782696 PMC4000604

[B25] Fromkin V. (1978).Tone: A Linguistic Survey. New York, NY: Academic Press.

[B26] Grosjean F. (2010). Bilingual. Cambridge; Massachusetts, MA; London; England: Harvard University Press 10.4159/9780674056459

[B27] Hallé P. A. Chang Y. C. Best C. T. (2004). Identification and discrimination of Mandarin Chinese tones by Mandarin Chinese vs. French listeners. J. Phon. 32, 395–421. 10.1016/S0095-4470(03)00016-0

[B28] Hao Y. C. (2012). Second language acquisition of Mandarin Chinese tones by tonal and non-tonal language speakers. J. Phon. 40, 269–279. 10.1016/j.wocn.2011.11.001

[B29] Hockley N. S. Polka L. (1994). A developmental study of audiovisual speech perception using the McGurk paradigm. J. Acoust. Soc. Am. 96, 3309–3309. 10.1121/1.410782

[B30] Horlyck S. Reid A. Burnham D. (2012). The relationship between learning to read and language-specific speech perception: maturation vs. experience. Sci. Stud. Read. 16, 218–239. 10.1080/10888438.2010.546460.

[B31] Jensen B. T. (2008). Metalinguistic Awareness. Encyclopedia of Bilingual Education. Thousand Oaks, CA: SAGE Publications.

[B32] Kasisopa B. Reilly R. Luksaneeyanawin S. Burnham D. (2016). Child readers' eye movements in reading Thai. Vis. Res. 123, 8–19. 10.1016/j.visres.2015.07.009 27137836

[B33] Kasisopa B. Reilly R. G. Luksaneeyanawin S. Burnham D. (2013). Eye movements while reading an unspaced writing system: the case of Thai. Vis. Res. 86, 71–80. 10.1016/j.visres.2013.04.007 23608059

[B34] Li C. N. Thompson S. A. (1977). The acquisition of tone in Mandarin-speaking children. J. Child Lang. 4, 185–199. 10.1017/S0305000900001598

[B35] Massaro D. W. (1984). Children's perception of visual and auditory speech. Child Dev. 55, 1777–1788. 10.2307/1129925 6510054

[B36] Massaro D. W. Thompson L. A. Barron B. Laren E. (1986). Developmental changes in visual and auditory contributions to speech perception. J. Exp. Child Psychol. 41, 93–113. 10.1016/0022-0965(86)90053-6 3950540

[B37] Mattock K. Burnham D. (2006). Chinese and English infants' tone perception: evidence for perceptual reorganization. Infancy 10, 241–265. 10.1207/s15327078in1003_3

[B38] Mattock K. Molnar M. Polka L. Burnham D. (2008). The developmental course of lexical tone perception in the first year of life. Cognition 106, 1367–1381. 10.1016/j.cognition.2007.07.002 17707789

[B39] McBride-Chang C. Shu H. Zhou A. Wat C. P. Wagner R. K. (2003). Morphological awareness uniquely predicts young children's Chinese character recognition. J. Educ. Psychol. 95, 743–751. 10.1037/0022-0663.95.4.743

[B40] McDougall S. Hulme C. Ellis A. Monk A. (1994). Learning to read: the role of short-term memory and phonological skills. J. Exp. Child Psychol. 58, 112–133. 10.1006/jecp.1994.1028 8064216

[B41] McGurk H. McDonald J. (1976). Hearing lips and seeing voices. Nature 264, 746–748. 10.1038/264746a0 1012311

[B42] Mixdorff H. Burnham D. Vignali G. Charnvivit P. (2005a). Are there facial correlates of Thai lexical tones?, in Proceedings of the 9th European Conference on Speech Communication and Technology. (Bonn: ISCA).

[B43] Mixdorff H. Charnvivit P. Burnham D. K. (2005b). Auditory-visual perception of syllabic tones in Thai, in Proceedings of the Auditory-Visual Speech Processing International Conference, 2005 eds Vatikiotis-Bateson E. Burnham D. Fels S. (Adelaide, SA: Causal Productions), 3–8.

[B44] Mixdorff H. Hu Y. Burnham D. (2005c). Visual cues in Mandarin tone perception, in Proceedings of the 9^th^ European Conference on Speech Communication and Technology. (Bonn, Germany: ISCA), 405–408.

[B45] Polka L. Werker J. F. (1994). Developmental changes in perception of nonnative vowel contrasts. J. Exp. Psychol. Hum. Percept. Perform. 20, 421–435. 10.1037/0096-1523.20.2.421 8189202

[B46] Rosenblum L. D. Schmuckler M. A. Johnson J. A. (1997). The McGurk effect in infants. Percept. Psychophys. 59, 347–357. 10.3758/BF03211902 9136265

[B47] Sekiyama K. Burnham D. (2008). Impact of language on development of auditory-visual speech perception. Dev. Sci. 11, 306–320. 10.1111/j.1467-7687.2008.00677.x 18333984

[B48] Sereno J. Maniwa K. (2006). Age contributions to language learning. Abstr. Psychon. Soc. 11, 4041–4109.

[B49] Sereno J. A. (2017). How category learning occurs in adults and children, in The Speech Processing Lexicon: Neurocognitive and Behavioural Approaches, eds Lahiri A. Kotzor S. (Berlin: Mouton de Gruyter), 192–209. 10.1515/9783110422658-010

[B50] Singh L. Foong J. (2012). Influences of lexical tone and pitch on word recognition in bilingual infants. Cognition 124, 128–142. 10.1016/j.cognition.2012.05.008 22682766 PMC3390932

[B51] Smith D. Burnham D. (2012). Facilitation of Mandarin tone perception by visual speech in clear and degraded audio: implications for cochlear implants. J. Acoust. Soc. Am. 131, 1480–1489. 10.1121/1.3672703 22352518

[B52] Tyler M. D. Burnham D. K. (2006). Orthographic influences on phoneme deletion response times. Q. J. Exp. Psychol. 59, 2010–2031. 10.1080/17470210500521828 16987786

[B53] Vatiktiotis-Bateson E. Kuratate T. Munhall K. G. Yehia H. C. (2000). The production and perception of a realistic talking face, in LP'98, Item order in language and speech eds Fujimura O. Joseph B. D. D. Palek B. (Prague: Charles University, Karolinum Press). 439–460.

[B54] Wang Y. Kuhl P. K. (2003). Evaluating the “critical period” hypothesis: Perceptual learning of Mandarin tones in American adults and American children at 6, 10, and 14 years of age, in Proceedings of the 15th International Congress of Phonetic Sciences (Barcelona), 1537–1540.

[B55] Wang Y. Spence M. M. Jongman A. Sereno J. A. (1999). Training American listeners to perceive Mandarin tones. J. Acoust. Soc. Am. 106, 3649–3658. 10.1121/1.428217 10615703

[B56] Wayland R. Guion S. (2003). Perceptual discrimination of Thai tones by naïve and experienced learners of Thai. Appl. Psycholinguist. 24, 113–129. 10.1017/S0142716403000067

[B57] Wayland R. P. Guion S. G. (2004). Training English and Chinese listeners to perceive Thai tones: a preliminary report. Lang. Learn. 54, 681–712. 10.1111/j.1467-9922.2004.00283.x

[B58] Werker J. F. Logan J. S. (1985). Cross-language evidence for three factors in speech perception. Percept. Psychophys. 37, 35–44. 10.3758/BF03207136 3991316

[B59] Werker J. F. Tees R. C. (1984). Cross-language speech perception: evidence for perceptual reorganization during the first year of life. Infant Behav. Dev. 7, 49–63. 10.1016/S0163-6383(84)80022-3

[B60] Wong P. Schwartz R. G. Jenkins J. J. (2005). Perception and production of lexical tones by 3-year-old, Mandarin-speaking children. J. Speech Lang. Hear. Res. 48, 1065–1079. 10.1044/1092-4388(2005/074) 16411796

[B61] Yeung H. H. Chen K. H. Werker J. F. (2013). When does native language input affect phonetic perception? The precocious case of lexical tone. J. Mem. Lang. 68, 123–139. 10.1016/j.jml.2012.09.004

[B62] Yip M. J. W. (2002). Tone. (New York, NY: Cambridge University Press), 1–14.

