# Supplementary material for: Training Children to Perceive Non-native Lexical Tones: Tone Language Background, Bilingualism, and Auditory-Visual Information
Source: Front Psychol. 2018 Sep 4;9:1508. doi: 10.3389/fpsyg.2018.01508 (PMC6131621; doi:10.3389/fpsyg.2018.01508)
Supplement: Supplementary file 1 [file Table_1.DOCX]

**Supplementary Material**

**Table A:** *Mandarin Syllables (in Pinyin form) used in Training Sessions*

| **Item No.** | **Training 1** | **Training 2** | **Training 3** | **Training 4** | **Training 5** | **Training 6** |
| --- | --- | --- | --- | --- | --- | --- |
| 1 | bei55 | ben55 | cen55 | che55 | chui55 | chun55 |
| 2 | cen35 | ceng35 | chui35 | chun35 | cuan35 | cong35 |
| 3 | dan214 | ding214 | dong214 | duan214 | dun214 | er214 |
| 4 | ben51 | bei51 | ceng51 | cu51 | che51 | cuan51 |
| 5 | cong55 | cu55 | die55 | dun55 | gang55 | gou55 |
| 6 | die35 | er35 | heng35 | hua35 | lai35 | lian35 |
| 7 | gong214 | gou214 | guan214 | juan214 | jing214 | ku214 |
| 8 | ding51 | dan51 | duan51 | dong51 | gong51 | gang51 |
| 9 | guan55 | he55 | jian55 | jing55 | jiu55 | kou55 |
| 10 | ling35 | mai35 | men35 | mian35 | min35 | nian35 |
| 11 | lian214 | lie214 | ling214 | lu214 | mai214 | mei214 |
| 12 | he51 | heng51 | hua51 | jian51 | juan51 | jiu51 |
| 13 | ku55 | men55 | lie55 | qiu55 | pan55 | que55 |
| 14 | nu35 | nong35 | nuo35 | pan35 | pei35 | qiu35 |
| 15 | min214 | nu214 | ran214 | rao214 | re214 | ren214 |
| 16 | kou51 | lai51 | lu51 | mei51 | nian51 | mian51 |
| 17 | sao55 | ta55 | sha55 | wo55 | xian55 | xun55 |
| 18 | que35 | ran35 | ren35 | rong35 | rou35 | ru35 |
| 19 | rong214 | ruan214 | sao214 | shua214 | shui214 | wo214 |
| 20 | nong51 | nuo51 | pei51 | re51 | rao51 | rou51 |
| 21 | zhen55 | zhong55 | zhou55 | zong55 | zuan55 | zu55 |
| 22 | ruan35 | shui35 | xian35 | xun35 | zu35 | zhou35 |
| 23 | xiu214 | zhen214 | zhong214 | zhua214 | zong214 | zuan214 |
| 24 | ru51 | sha51 | shua51 | ta51 | xiu51 | zhua51 |
| 25 | bei55 | ben55 | cen55 | che55 | chui55 | chun55 |
| 26 | cen35 | ceng35 | chui35 | chun35 | cuan35 | cong35 |
| 27 | dan214 | ding214 | dong214 | duan214 | dun214 | er214 |
| 28 | ben51 | bei51 | ceng51 | cu51 | che51 | cuan51 |
| 29 | cong55 | cu55 | die55 | dun55 | gang55 | gou55 |
| 30 | die35 | er35 | heng35 | hua35 | lai35 | lian35 |
| 31 | gong214 | gou214 | guan214 | juan214 | jing214 | ku214 |
| 32 | ding51 | dan51 | duan51 | dong51 | gong51 | gang51 |
| 33 | guan55 | he55 | jian55 | jing55 | jiu55 | kou55 |
| 34 | ling35 | mai35 | men35 | mian35 | min35 | nian35 |
| 35 | lian214 | lie214 | ling214 | lu214 | mai214 | mei214 |
| 36 | he51 | heng51 | hua51 | jian51 | juan51 | jiu51 |
| 37 | ku55 | men55 | lie55 | qiu55 | pan55 | que55 |
| 38 | nu35 | nong35 | nuo35 | pan35 | pei35 | qiu35 |
| 39 | min214 | nu214 | ran214 | rao214 | re214 | ren214 |
| 40 | kou51 | lai51 | lu51 | mei51 | nian51 | mian51 |
| 41 | sao55 | ta55 | sha55 | wo55 | xian55 | xun55 |
| 42 | que35 | ran35 | ren35 | rong35 | rou35 | ru35 |
| 43 | rong214 | ruan214 | sao214 | shua214 | shui214 | wo214 |
| 44 | nong51 | nuo51 | pei51 | re51 | rao51 | rou51 |
| 45 | zhen55 | zhong55 | zhou55 | zong55 | zuan55 | zu55 |
| 46 | ruan35 | shui35 | xian35 | xun35 | zu35 | zhou35 |
| 47 | xiu214 | zhen214 | zhong214 | zhua214 | zong214 | zuan214 |
| 48 | ru51 | sha51 | shua51 | ta51 | xiu51 | zhua51 |
| 49 | bei55 | ben55 | cen55 | che55 | chui55 | chun55 |
| 50 | cen35 | ceng35 | chui35 | chun35 | cuan35 | cong35 |
| 51 | dan214 | ding214 | dong214 | duan214 | dun214 | er214 |
| 52 | ben51 | bei51 | ceng51 | cu51 | che51 | cuan51 |
| 53 | cong55 | cu55 | die55 | dun55 | gang55 | gou55 |
| 54 | die35 | er35 | heng35 | hua35 | lai35 | lian35 |
| 55 | gong214 | gou214 | guan214 | juan214 | jing214 | ku214 |
| 56 | ding51 | dan51 | duan51 | dong51 | gong51 | gang51 |
| 57 | guan55 | he55 | jian55 | jing55 | jiu55 | kou55 |
| 58 | ling35 | mai35 | men35 | mian35 | min35 | nian35 |
| 59 | lian214 | lie214 | ling214 | lu214 | mai214 | mei214 |
| 60 | he51 | heng51 | hua51 | jian51 | juan51 | jiu51 |
| 61 | ku55 | men55 | lie55 | qiu55 | pan55 | que55 |
| 62 | nu35 | nong35 | nuo35 | pan35 | pei35 | qiu35 |
| 63 | min214 | nu214 | ran214 | rao214 | re214 | ren214 |
| 64 | kou51 | lai51 | lu51 | mei51 | nian51 | mian51 |
| 65 | sao55 | ta55 | sha55 | wo55 | xian55 | xun55 |
| 66 | que35 | ran35 | ren35 | rong35 | rou35 | ru35 |
| 67 | rong214 | ruan214 | sao214 | shua214 | shui214 | wo214 |
| 68 | nong51 | nuo51 | pei51 | re51 | rao51 | rou51 |
| 69 | zhen55 | zhong55 | zhou55 | zong55 | zuan55 | zu55 |
| 70 | ruan35 | shui35 | xian35 | xun35 | zu35 | zhou35 |
| 71 | xiu214 | zhen214 | zhong214 | zhua214 | zong214 | zuan214 |
| 72 | ru51 | sha51 | shua51 | ta51 | xiu51 | zhua51 |
| 73 | bei55 | ben55 | cen55 | che55 | chui55 | chun55 |
| 74 | cen35 | ceng35 | chui35 | chun35 | cuan35 | cong35 |
| 75 | dan214 | ding214 | dong214 | duan214 | dun214 | er214 |
| 76 | ben51 | bei51 | ceng51 | cu51 | che51 | cuan51 |
| 77 | cong55 | cu55 | die55 | dun55 | gang55 | gou55 |
| 78 | die35 | er35 | heng35 | hua35 | lai35 | lian35 |
| 79 | gong214 | gou214 | guan214 | juan214 | jing214 | ku214 |
| 80 | ding51 | dan51 | duan51 | dong51 | gong51 | gang51 |
| 81 | guan55 | he55 | jian55 | jing55 | jiu55 | kou55 |
| 82 | ling35 | mai35 | men35 | mian35 | min35 | nian35 |
| 83 | lian214 | lie214 | ling214 | lu214 | mai214 | mei214 |
| 84 | he51 | heng51 | hua51 | jian51 | juan51 | jiu51 |
| 85 | ku55 | men55 | lie55 | qiu55 | pan55 | que55 |
| 86 | nu35 | nong35 | nuo35 | pan35 | pei35 | qiu35 |
| 87 | min214 | nu214 | ran214 | rao214 | re214 | ren214 |
| 88 | kou51 | lai51 | lu51 | mei51 | nian51 | mian51 |
| 89 | sao55 | ta55 | sha55 | wo55 | xian55 | xun55 |
| 90 | que35 | ran35 | ren35 | rong35 | rou35 | ru35 |
| 91 | rong214 | ruan214 | sao214 | shua214 | shui214 | wo214 |
| 92 | nong51 | nuo51 | pei51 | re51 | rao51 | rou51 |
| 93 | zhen55 | zhong55 | zhou55 | zong55 | zuan55 | zu55 |
| 94 | ruan35 | shui35 | xian35 | xun35 | zu35 | zhou35 |
| 95 | xiu214 | zhen214 | zhong214 | zhua214 | zong214 | zuan214 |
| 96 | ru51 | sha51 | shua51 | ta51 | xiu51 | zhua51 |

**Table B:** *Percent correct of Pre-test (Pre-), Post-test (Post-), Post minus Pre (Post-Pre), and performance gain (gain) scores for all participants*.

|  |  | **AO Training** | | | | | | | | **AV Training** | | | | | | | |
| --- | --- | --- | --- | --- | --- | --- | --- | --- | --- | --- | --- | --- | --- | --- | --- | --- | --- |
|  |  | **ao Test** | | | | **av Test** | | | | **ao Test** | | | | **av Test** | | | |
| **Age** | **Language** | ***Pre-*** | ***Post-*** | ***Post-Pre*** | ***Gain*** | ***Pre-*** | ***Post-*** | ***Post-Pre*** | ***Gain*** | ***Pre-*** | ***Post-*** | ***Post-Pre*** | ***Gain*** | ***Pre-*** | ***Post-*** | ***Post-Pre*** | ***Gain*** |
| **6** | **Mono-Eng** | 20 | 23 | 3 | **16** | 18 | 21 | 3 | **18** | 17 | 28 | 11 | **69** | 26 | 19 | -7 | **-28** |
|  | **Mono-Eng** | 25 | 42 | 17 | **67** | 26 | 24 | -2 | **-8** | 21 | 31 | 10 | **50** | 26 | 25 | -1 | **-4** |
|  | **Mono-Eng** | 25 | 31 | 6 | **25** | 28 | 43 | 15 | **52** | 27 | 28 | 1 | **4** | 27 | 25 | -2 | **-8** |
|  | **Mono-Eng** | 27 | 24 | -3 | **-12** | 32 | 26 | -6 | **-19** | 29 | 46 | 17 | **57** | 45 | 38 | -7 | **-16** |
|  | **Mono-Thai** | 23 | 18 | -5 | **-23** | 23 | 31 | 8 | **36** | 20 | 25 | 5 | **26** | 19 | 22 | 3 | **17** |
|  | **Mono-Thai** | 31 | 28 | -3 | **-10** | 24 | 17 | -7 | **-30** | 20 | 27 | 7 | **37** | 27 | 21 | -6 | **-23** |
|  | **Mono-Thai** | 32 | 22 | -10 | **-32** | 38 | 27 | -10 | **-28** | 41 | 26 | -15 | **-36** | 27 | 23 | -4 | **-15** |
|  | **Mono-Thai** | 34 | 23 | -11 | **-33** | 48 | 23 | -25 | **-52** | 43 | 26 | -17 | **-39** | 34 | 24 | -10 | **-30** |
|  | **Bi-Eng/Arabic** | 15 | 29 | 15 | **100** | 16 | 19 | 3 | **20** | 15 | 30 | 16 | **107** | 18 | 19 | 1 | **6** |
|  | **Bi-Eng/Arabic** | 19 | 38 | 19 | **100** | 24 | 27 | 3 | **13** | 22 | 22 | 0 | **0** | 23 | 21 | -2 | **-9** |
|  | **Bi-Eng/Arabic** | 24 | 25 | 1 | **4** | 26 | 29 | 3 | **12** | 25 | 28 | 3 | **13** | 28 | 35 | 7 | **26** |
|  | **Bi-Eng/Arabic** | 25 | 34 | 9 | **38** | 27 | 23 | -4 | **-15** | 26 | 15 | -11 | **-44** | 30 | 23 | -7 | **-24** |
|  | **Bi-Eng/Arabic** | 25 | 26 | 1 | **4** | 30 | 25 | -5 | **-17** | 31 | 14 | -18 | **-57** | 31 | 31 | 0 | **0** |
|  | **Bi-Eng/Arabic** | 27 | 42 | 15 | **54** | 31 | 26 | -5 | **-17** | 33 | 29 | -4 | **-12** | 32 | 20 | -13 | **-39** |
|  | **Bi-Eng/Thai** | 22 | 45 | 23 | **105** | 21 | 25 | 4 | **20** | 23 | 26 | 3 | **14** | 20 | 21 | 1 | **5** |
|  | **Bi-Eng/Thai** | 24 | 26 | 2 | **9** | 22 | 29 | 7 | **33** | 26 | 22 | -4 | **-16** | 21 | 21 | 0 | **0** |
|  | **Bi-Eng/Thai** | 25 | 22 | -3 | **-13** | 24 | 18 | -6 | **-26** | 28 | 26 | -2 | **-7** | 22 | 25 | 3 | **14** |
|  | **Bi-Eng/Thai** | 26 | 24 | -2 | **-8** | 26 | 26 | 0 | **0** | 29 | 32 | 3 | **11** | 25 | 27 | 2 | **8** |
|  | **Bi-Eng/Thai** | 28 | 25 | -3 | **-11** | 28 | 28 | 0 | **0** | 30 | 25 | -5 | **-17** | 25 | 27 | 2 | **8** |
|  | **Bi-Eng/Thai** | 30 | 23 | -7 | **-24** | 32 | 39 | 6 | **19** | 33 | 21 | -13 | **-38** | 25 | 23 | -2 | **-8** |
| **8** | **Mono-Eng** | 21 | 15 | -6 | **-30** | 18 | 28 | 10 | **59** | 24 | 23 | -1 | **-4** | 26 | 31 | 5 | **20** |
|  | **Mono-Eng** | 24 | 24 | 0 | **0** | 19 | 24 | 5 | **28** | 25 | 30 | 5 | **21** | 27 | 27 | 0 | **0** |
|  | **Mono-Eng** | 26 | 38 | 11 | **44** | 23 | 27 | 4 | **18** | 26 | 26 | 0 | **0** | 27 | 28 | 1 | **4** |
|  | **Mono-Eng** | 27 | 23 | -4 | **-15** | 27 | 23 | -4 | **-15** | 28 | 27 | -1 | **-4** | 28 | 25 | -3 | **-11** |
|  | **Mono-Eng** | 29 | 23 | -6 | **-21** | 35 | 33 | -2 | **-6** |  |  |  |  |  |  |  |  |
|  | **Mono-Thai** | 18 | 22 | 4 | **24** | 14 | 27 | 14 | **100** | 21 | 48 | 27 | **130** | 21 | 26 | 5 | **25** |
|  | **Mono-Thai** | 19 | 22 | 3 | **17** | 21 | 28 | 7 | **35** | 23 | 59 | 36 | **159** | 27 | 70 | 43 | **158** |
|  | **Mono-Thai** | 33 | 41 | 7 | **22** | 24 | 39 | 15 | **61** | 27 | 26 | -1 | **-4** | 29 | 54 | 25 | **86** |
|  | **Mono-Thai** | 44 | 25 | -19 | **-43** | 43 | 25 | -18 | **-41** | 42 | 50 | 8 | **20** | 44 | 54 | 10 | **24** |
|  | **Bi-Eng/Arabic** | 18 | 23 | 5 | **29** | 20 | 26 | 6 | **32** | 22 | 22 | 0 | **0** | 17 | 23 | 6 | **37** |
|  | **Bi-Eng/Arabic** | 23 | 17 | -6 | **-27** | 22 | 27 | 5 | **24** | 23 | 20 | -3 | **-14** | 22 | 25 | 3 | **14** |
|  | **Bi-Eng/Arabic** | 24 | 26 | 2 | **9** | 24 | 28 | 4 | **17** | 24 | 23 | -1 | **-4** | 25 | 19 | -6 | **-25** |
|  | **Bi-Eng/Arabic** | 25 | 26 | 1 | **4** | 24 | 33 | 9 | **39** | 24 | 25 | 1 | **4** | 27 | 25 | -2 | **-8** |
|  | **Bi-Eng/Arabic** | 27 | 26 | -1 | **-4** | 25 | 29 | 4 | **17** | 25 | 26 | 1 | **4** | 30 | 25 | -5 | **-17** |
|  | **Bi-Eng/Arabic** | 33 | 24 | -9 | **-28** | 27 | 25 | -2 | **-8** | 29 | 32 | 3 | **11** | 30 | 32 | 2 | **7** |
|  | **Bi-Eng/Thai** | 10 | 46 | 35 | **340** | 17 | 39 | 22 | **131** | 16 | 42 | 26 | **167** | 24 | 21 | -3 | **-13** |
|  | **Bi-Eng/Thai** | 19 | 29 | 10 | **56** | 19 | 33 | 15 | **78** | 21 | 27 | 6 | **30** | 25 | 20 | -5 | **-21** |
|  | **Bi-Eng/Thai** | 21 | 23 | 2 | **10** | 22 | 25 | 3 | **14** | 21 | 28 | 7 | **35** | 26 | 26 | 0 | **0** |
|  | **Bi-Eng/Thai** | 21 | 31 | 10 | **50** | 27 | 22 | -5 | **-19** | 21 | 25 | 4 | **20** | 26 | 22 | -4 | **-16** |
|  | **Bi-Eng/Thai** | 24 | 42 | 18 | **74** | 30 | 30 | 0 | **0** | 25 | 65 | 40 | **158** | 28 | 60 | 32 | **115** |
|  | **Bi-Eng/Thai** | 26 | 42 | 16 | **60** | 32 | 41 | 8 | **26** | 26 | 24 | -2 | **-8** | 28 | 38 | 9 | **33** |

**Table C:** *Mean Raw Accuracy Scores (and SD) and Performance Gain for each Mandarin tone of each group.*

| **Group** | **Bi-Eng/Arabic** | | | | | | | | | | | |
| --- | --- | --- | --- | --- | --- | --- | --- | --- | --- | --- | --- | --- |
| **Age** | **6yo** | | | | | | **8yo** | | | | | |
| **Mode** | **AO** | | | **AV** | | | **AO** | | | **AV** | | |
| **Test** | ***Pre-test*** | ***Post-test*** | ***Gain(%)*** | ***Pre-test*** | ***Post-test*** | ***Gain(%)*** | ***Pre-test*** | ***Post-test*** | ***Gain(%)*** | ***Pre-test*** | ***Post-test*** | ***Gain(%)*** |
| **Tone 55** | 0.24 *(0.43)* | 0.40 *(0.49)* | 64.29 | 0.25 *(0.43)* | 0.42 *(0.49)* | 68.06 | 0.25 *(0.44)* | 0.21 *(0.41)* | -16.44 | 0.19 *(0.39)* | 0.23 *(0.42)* | 18.18 |
| **Tone 35** | 0.25 *(0.43)* | 0.27 *(0.45)* | 8.33 | 0.27 *(0.44)* | 0.22 *(0.42)* | -16.88 | 0.30 *(0.46)* | 0.24 *(0.43)* | -18.60 | 0.26 *(0.44)* | 0.29 *(0.46)* | 13.51 |
| **Tone214** | 0.23 *(0.42)* | 0.20 *(0.40)* | -9.23 | 0.28 *(0.45)* | 0.18 *(0.39)* | -34.57 | 0.19 *(0.39)* | 0.29 *(0.46)* | 55.56 | 0.27 *(0.45)* | 0.30 *(0.46)* | 7.59 |
| **Tone 51** | 0.24 *(0.43)* | 0.23 *(0.42)* | -2.94 | 0.26 *(0.44)* | 0.17 *(0.37)* | -35.14 | 0.25 *(0.43)* | 0.22 *(0.41)* | -13.89 | 0.26 *(0.44)* | 0.28 *(0.45)* | 8.11 |

| **Group** | **Bi-Eng/Thai** | | | | | | | | | | | |
| --- | --- | --- | --- | --- | --- | --- | --- | --- | --- | --- | --- | --- |
| **Age** | **6yo** | | | | | | **8yo** | | | | | |
| **Mode** | **AO** | | | **AV** | | | **AO** | | | **AV** | | |
| **Test** | ***Pre-test*** | ***Post-test*** | ***Gain(%)*** | ***Pre-test*** | ***Post-test*** | ***Gain(%)*** | ***Pre-test*** | ***Post-test*** | ***Gain(%)*** | ***Pre-test*** | ***Post-test*** | ***Gain(%)*** |
| **Tone 55** | 0.27 *(0.45)* | 0.32 *(0.47)* | 16.46 | 0.23 *(0.42)* | 0.27 *(0.44)* | 16.67 | 0.18 *(0.38)* | 0.35 *(0.48)* | 96.08 | 0.22 *(0.42)* | 0.30 *(0.46)* | 35.94 |
| **Tone 35** | 0.34 *(0.47)* | 0.27 *(0.44)* | -20.62 | 0.31 *(0.46)* | 0.33 *(0.47)* | 4.44 | 0.24 *(0.43)* | 0.38 *(0.49)* | 57.97 | 0.36 *(0.48)* | 0.39 *(0.49)* | 5.71 |
| **Tone214** | 0.24 *(0.43)* | 0.26 *(0.44)* | 11.76 | 0.23 *(0.42)* | 0.24 *(0.43)* | 4.55 | 0.21 *(0.41)* | 0.42 *(0.49)* | 96.72 | 0.26 *(0.44)* | 0.44 *(0.50)* | 70.27 |
| **Tone 51** | 0.23 *(0.42)* | 0.19 *(0.39)* | -18.18 | 0.20 *(0.40)* | 0.18 *(0.39)* | -7.02 | 0.24 *(0.43)* | 0.33 *(0.47)* | 39.71 | 0.19 *(0.40)* | 0.22 *(0.41)* | 10.71 |

| **Group** | **Mono-Eng** | | | | | | | | | | | |
| --- | --- | --- | --- | --- | --- | --- | --- | --- | --- | --- | --- | --- |
| **Age** | **6yo** | | | | | | **8yo** | | | | | |
| **Mode** | **AO** | | | **AV** | | | **AO** | | | **AV** | | |
| **Test** | ***Pre-test*** | ***Post-test*** | ***Gain(%)*** | ***Pre-test*** | ***Post-test*** | ***Gain(%)*** | ***Pre-test*** | ***Post-test*** | ***Gain(%)*** | ***Pre-test*** | ***Post-test*** | ***Gain(%)*** |
| **Tone 55** | 0.28 *(0.45)* | 0.41 *(0.49)* | 45.90 | 0.33 *(0.47)* | 0.40 *(0.49)* | 20.83 | 0.29 *(0.45)* | 0.29 *(0.46)* | 1.61 | 0.29 *(0.46)* | 0.34 *(0.47)* | 15.87 |
| **Tone 35** | 0.25 *(0.44)* | 0.30 *(0.46)* | 18.18 | 0.25 *(0.44)* | 0.26 *(0.44)* | 3.64 | 0.31 *(0.46)* | 0.30 *(0.46)* | -1.52 | 0.27 *(0.45)* | 0.26 *(0.44)* | -3.39 |
| **Tone214** | 0.28 *(0.45)* | 0.29 *(0.46)* | 3.28 | 0.35 *(0.48)* | 0.25 *(0.44)* | -27.63 | 0.25 *(0.44)* | 0.25 *(0.43)* | -1.82 | 0.27 *(0.45)* | 0.28 *(0.45)* | 1.69 |
| **Tone 51** | 0.16 *(0.37)* | 0.23 *(0.42)* | 42.86 | 0.20 *(0.40)* | 0.22 *(0.42)* | 11.63 | 0.17 *(0.38)* | 0.21 *(0.41)* | 21.62 | 0.21 *(0.41)* | 0.18 *(0.38)* | -15.56 |

| **Group** | **Mono-Thai** | | | | | | | | | | | |
| --- | --- | --- | --- | --- | --- | --- | --- | --- | --- | --- | --- | --- |
| **Age** | **6yo** | | | | | | **8yo** | | | | | |
| **Mode** | **AO** | | | **AV** | | | **AO** | | | **AV** | | |
| **Test** | ***Pre-test*** | ***Post-test*** | ***Gain(%)*** | ***Pre-test*** | ***Post-test*** | ***Gain(%)*** | ***Pre-test*** | ***Post-test*** | ***Gain(%)*** | ***Pre-test*** | ***Post-test*** | ***Gain(%)*** |
| **Tone 55** | 0.50 *(0.50)* | 0.18 *(0.38)* | -64.58 | 0.52 *(0.50)* | 0.16 *(0.36)* | -70.00 | 0.66 *(0.48)* | 0.43 *(0.50)* | -34.92 | 0.61 *(0.49)* | 0.47 *(0.50)* | -23.08 |
| **Tone 35** | 0.29 *(0.46)* | 0.22 *(0.42)* | -23.21 | 0.28 *(0.45)* | 0.19 *(0.40)* | -30.19 | 0.16 *(0.36)* | 0.27 *(0.45)* | 73.33 | 0.17 *(0.37)* | 0.32 *(0.47)* | 90.63 |
| **Tone214** | 0.31 *(0.46)* | 0.24 *(0.43)* | -20.34 | 0.26 *(0.44)* | 0.23 *(0.42)* | -8.16 | 0.14 *(0.34)* | 0.33 *(0.47)* | 142.31 | 0.18 *(0.38)* | 0.36 *(0.48)* | 102.94 |
| **Tone 51** | 0.17 *(0.37)* | 0.33 *(0.47)* | 96.88 | 0.15 *(0.35)* | 0.35 *(0.48)* | 142.86 | 0.18 *(0.39)* | 0.44 *(0.50)* | 140.00 | 0.16 *(0.36)* | 0.47 *(0.50)* | 200.00 |
